# Inhibiting LXRα phosphorylation in hematopoietic cells reduces inflammation and attenuates atherosclerosis and obesity in mice

**DOI:** 10.1038/s42003-021-01925-5

**Published:** 2021-03-26

**Authors:** Maud Voisin, Elina Shrestha, Claire Rollet, Cyrus A. Nikain, Tatjana Josefs, Mélanie Mahé, Tessa J. Barrett, Hye Rim Chang, Rachel Ruoff, Jeffrey A. Schneider, Michela L. Garabedian, Chris Zoumadakis, Chi Yun, Bara Badwan, Emily J. Brown, Adam C. Mar, Robert J. Schneider, Ira J. Goldberg, Inés Pineda-Torra, Edward A. Fisher, Michael J. Garabedian

**Affiliations:** 1 Department of Microbiology, NYU School of Medicine, New York, NY USA; 2 Division of Cardiology, Marc and Ruti Bell Program in Vascular Biology, Department of Medicine, NYU School of Medicine, New York, NY USA; 3 Division of Endocrinology, Department of Medicine, NYU School of Medicine, New York, NY USA; 4 Ordaos, Inc, New York, NY USA; 5 Department of Neuroscience and Physiology, NYU School of Medicine, New York, NY USA; 6 Neuroscience Institute, New York University Medical Center, New York, NY USA; 7 Centre for Cardiometabolic and Vascular Science, University College of London, London, UK

**Keywords:** Gene regulation in immune cells, Cardiovascular biology

## Abstract

Atherosclerosis and obesity share pathological features including inflammation mediated by innate and adaptive immune cells. LXRα plays a central role in the transcription of inflammatory and metabolic genes. LXRα is modulated by phosphorylation at serine 196 (LXRα pS196), however, the consequences of LXRα pS196 in hematopoietic cell precursors in atherosclerosis and obesity have not been investigated. To assess the importance of LXRα phosphorylation, bone marrow from LXRα WT and S196A mice was transplanted into *Ldlr*^*−/−*^ mice, which were fed a western diet prior to evaluation of atherosclerosis and obesity. Plaques from S196A mice showed reduced inflammatory monocyte recruitment, lipid accumulation, and macrophage proliferation. Expression profiling of CD68^+^ and T cells from S196A mouse plaques revealed downregulation of pro-inflammatory genes and in the case of CD68^+^ upregulation of mitochondrial genes characteristic of anti-inflammatory macrophages. Furthermore, S196A mice had lower body weight and less visceral adipose tissue; this was associated with transcriptional reprograming of the adipose tissue macrophages and T cells, and resolution of inflammation resulting in less fat accumulation within adipocytes. Thus, reducing LXRα pS196 in hematopoietic cells attenuates atherosclerosis and obesity by reprogramming the transcriptional activity of LXRα in macrophages and T cells to promote an anti-inflammatory phenotype.

## Abstract (web summary)

Voisin et al. show that reducing phosphorylation at serine 196 of LXRα (LXRα pS196) in hematopoietic cells attenuates atherosclerosis and obesity of mice fed a high-fat diet. They find that the transcriptional activity of LXRα is reprogrammed in macrophages and T cells to promote an anti-inflammatory phenotype, thus identifying a functional role of LXRα pS196 in immune cells.

## Introduction

LXRα is a desmosterol- and oxysterol-activated transcription factor that controls the expression of genes involved in cholesterol homeostasis, apoptosis, inflammation, and cell movement^1,2^. Activation of LXRα inhibits atherosclerosis progression in mouse models, which depends in part on LXRα activity in macrophages^3,4^. During atherogenesis, immune cells—mostly cholesterol-loaded macrophages accumulate in the arterial wall and form plaques^5–9^. Obesity, like atherosclerosis, is associated with lipid accumulation and chronic inflammation^10^. Macrophages accumulate in the visceral adipose tissue (VAT) of animals after being fed a western diet^11,12^, and produce inflammatory cytokines that contribute to metabolic dysfunction^13^.

We and others have shown that LXRα’s ability to activate transcription is modulated by phosphorylation at serine 196 (S196 in mouse LXRα and S198 in human LXRα), which significantly modifies its target gene repertoire^14–18^. Whereas LXRα pS196 promotes an inflammatory gene signature, unphosphorylated LXRα stimulates an anti-inflammatory gene expression profile. Consistent with this idea, in mouse models we observed an increase in LXRα pS196 in plaque macrophages under inflammatory conditions associated with atherosclerosis progression and a decrease in LXRα pS196 during the resolution of inflammation in regressing plaques^16,19^. Cholesterol-loaded macrophages and hepatocytes also showed increased LXRα pS196^15,20^. Thus, the lack of LXRα phosphorylation at S196 appears associated with an anti-inflammatory phenotype.

In this study, we examined the impact of LXRα pS196 in immune cells on atherosclerosis progression and obesity, using a bone marrow transplant approach from wild type (WT) and LXRα S196A knock-in mice, which are unable to be phosphorylated and should retain only the anti-inflammatory actions of LXRα. This approach has the potential to uncover the importance of LXRα phosphorylation activities within immune cells, while avoiding confounding effects of LXRα S196A expression in other organs. This strategy can also provide insight into potential communication between immune cell types when contrasted with effects on atherosclerosis and obesity from, for example, myeloid-specific LXRα S196A expression. Characterizing the role of LXRα pS196 in hematopoietic cells and their contribution to atherosclerosis and obesity has the potential to reveal new therapeutic strategies for both pathologies.

## Results

### LXRα S196A promotes an atheroprotective lipoprotein phenotype in a bone marrow transplant model in *Ldlr*^*−/−*^ mice

**Fig. 1:**
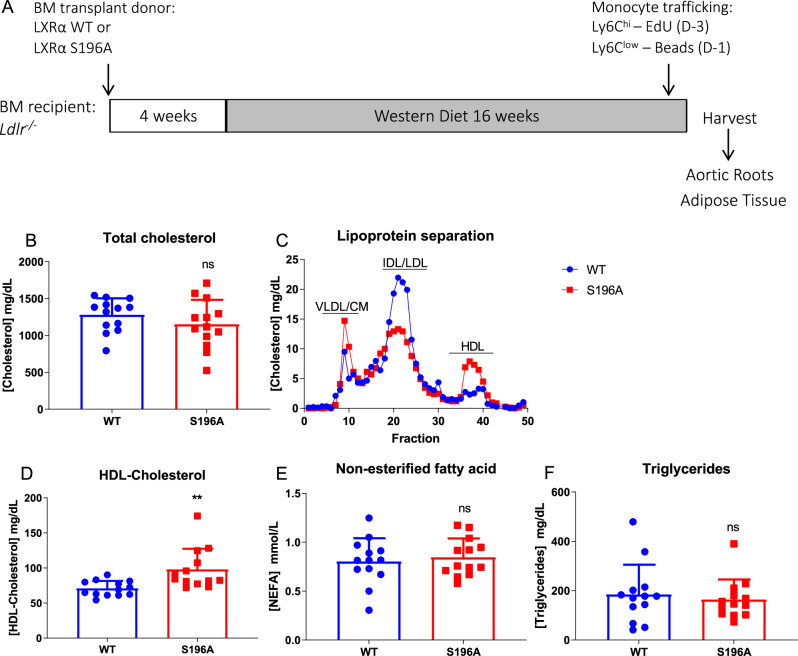
LXRα S196A increases HDL-C and decreases LDL-C levels in plasma. **A** Schematic of the bone marrow (BM) transplant experimental design. **B** Plasma total cholesterol levels from LXRα WT and S196A mice. **C** Plasma lipoproteins distribution as measured by FPLC chromatography between LXRα WT and S196A mice. VLDL: very low-density lipoprotein; CM: Chylomicron; IDL: intermediate-density lipoprotein; LDL: low-density lipoprotein; HDL: high-density lipoprotein. **D** HDL-Cholesterol measured by ELISA. **E** Plasma non-esterified fatty acids and **F** triglycerides concentrations in LXRα WT and S196A mice. Data are expressed as mean ± SD (*n* = 13 per group) and obtained from independent samples. *T* test; **P < 0.01.

Given the role that LXRα plays in cholesterol homeostasis and control of inflammation, we hypothesized that reducing LXRα pS196 would decrease inflammation and reduce atherosclerosis progression. Thus, we used an LXRα phosphorylation-deficient knock-in mouse, LXRα S196A, in a bone marrow transplantation model^14^. This approach allowed us to interrogate the requirement of LXRα phosphorylation on the entire cadre of immune cells expressing S196A compared to WT. LXRα S196A or WT mice were used as donors for bone marrow transplant into *Ldlr*^*−/−*^ recipients. Following reconstitution, mice were fed a western diet (42% kcal fat and 0.3% kcal cholesterol) for 16 weeks after which time atherogenesis was assessed (Fig. 1A). LXRα S196A did not alter the levels of total cholesterol (WT:1280.5 ± 224.3, S196A:1152.5 ± 331.1 mg/dL, *p* = 0.2599, Fig. 1B). After lipoprotein separation by fast protein liquid chromatography, S196A mice showed decreased atherogenic IDL/LDL cholesterol (LDL-C)^21^ and increased atheroprotective HDL cholesterol (HDL-C) compared to controls (Fig. 1C). We confirmed this increase by directly measuring HDL-C concentration in the plasma (WT:70.88 ± 10.78, S196A:98.09 ± 29.27 mg/dL, *p* = 0.0044, Fig. 1D). Nonesterified fatty acid (NEFA WT:0.804 ± 0.237, S196A:0.846 ± 0.194 mmol/L, *p* = 0.6207) and triglyceride (TG WT:184.5 ± 120.7, S196A:164.1 ± 81.15 mg/dL, *p* = 0.6174) levels in plasma were not changed by LXRα S196A (Fig. 1E, F). Similarly, hepatic lipid droplet accumulation was comparable in LXRα S196A and WT mice (WT:94.68 ± 23.93, S196A:87.59 ± 11.90 µm^2^, *p* = 0.6255; Supplementary Fig. S1A, B). To probe why the level of HDL-C is higher and LDL-C is lower in LXR S196A compared to WT we measured in liver and intestine the expression of genes involved in cholesterol homeostasis including cholesterol secretion (*Abca1, Abcg1*), cholesterol excretion (*Abcg5, Abcg8*), fatty acid metabolism (*Srebp1c*), fatty acid uptake (*Cd36*), cholesterol biosynthesis (*Hmgcor*), lipoprotein uptake (*Vldlr*, *Ldlr*), and intestinal cholesterol uptake (*Npc1l1*) (Supplementary Fig. S1C, D). None of these genes, with the exception of *Ldlr* in the liver, showed a significant change in expression between LXRα S196A and WT mice. Another possibility for the reduction in LDL-C could be an increase in the number Kupffer cells (KC) in the liver of LXRα S196A given the ability of KC to clear plasma LDL-C, the capacity of bone marrow-derived monocytes to give rise to KCs^22^, and the link between LXRα and KC ontogeny^23–25^. To measure the number of KC, we performed immunohistochemistry for Clec4f, a specific marker of KC from livers of LXRα S196A and WT mice. We observed a 60% increase in number of KC in the liver of LXRα S196A compared to WT (WT:9.83 ± 2.30, S196A: 16.05 ± 3.47% positive cells, *p* = 0.0044; Supplementary Fig. S1E, F). This increase is consistent with the reduction in LDL-C in mice expressing LXRα S196A, although additional functional studies, such as KC depletion, would be needed to determine the contribution of KCs to lipid homeostasis.

Differences in hepatic TG and cholesterol levels, or the secretion of triglyceride-rich lipoproteins from the liver as well as differences in TG absorption from the intestine could also contribute to the changes in the lipoprotein profiles in mice expressing LXRα S196A. To assess this, we measured hepatic cholesterol and TG levels in LXRα S196A and WT mice, hepatic VLDL-TG secretion on poloxamer 407 treated mice [a potent inhibitor of Lipoprotein lipase], as well as intestinal absorption of TG after plasma triglyceride levels were transiently increased by oral gavage of olive oil in full-body knock-in LXRα S196A and WT mice fed on chow diet (Supplementary Fig. S2A–C). While there was no change in hepatic cholesterol (WT: 40.6 ± 6.6, S196A:36.8 ± 1.6 mg/g liver, *p* = 0.1982), there was a reduction in hepatic TG in LXRα S196A mice compared to control (WT: 157.8 ± 32.5, S196A:121.5 ± 20.9 mg/g liver, *p* = 0.0439; Supplementary Fig. S2A). We observed an increase in VLDL secretion from the liver (2 h WT:396,6 ± 131.1, S196A: 727.1 ± 177.3 mg/dL, *p* = 0.0228; 4 h WT:1410.4 ± 487.6, S196A:2146.9 ± 206.7 mg/dL, *p* = 0.0098; Supplementary Fig. S2B), and increased clearance of TG from the intestine in LXRα S196A mice compared to WT (2 h WT:107.4 ± 26.0, S196A:52.8 ± 15.5 mg/dL, *p* = 0.0056, Supplementary Fig. S2C). These findings suggest that the atheroprotective lipoprotein profile in LXRα S196A mice is multifactorial and reflects increased KC, increased TG export from the liver, and enhanced TG elimination by the intestine.

### LXRα S196A expressed in bone marrow-derived cells affects multiple kinetic factors governing plaque macrophage content

**Fig. 2:**
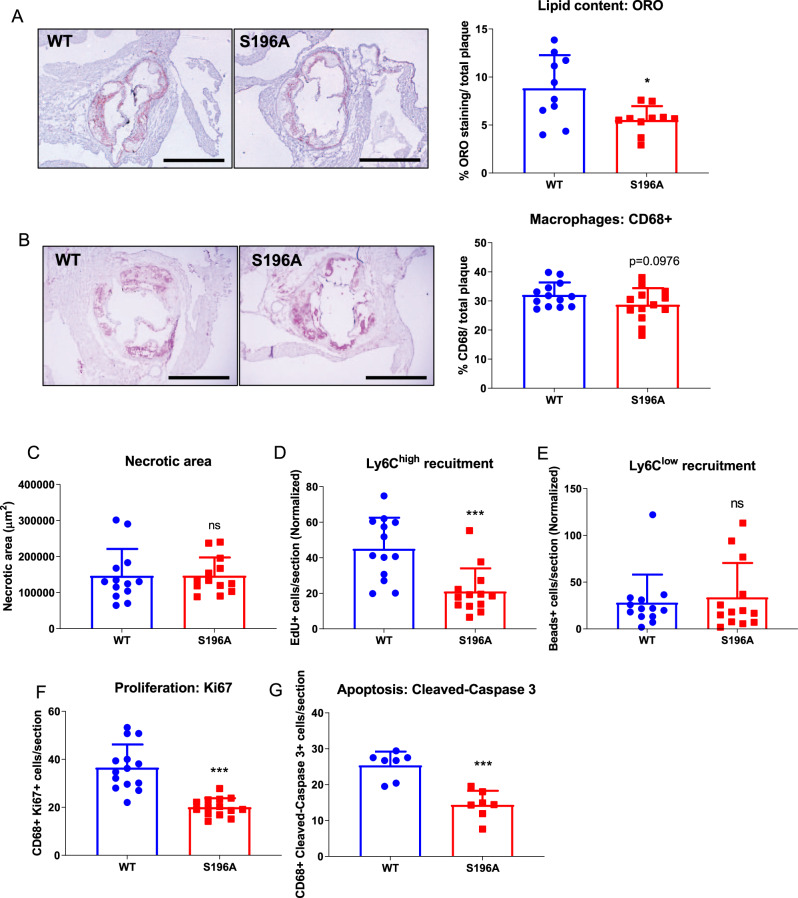
LXRα S196A decreases lipid accumulation in plaque macrophages, reduces monocyte recruitment and macrophage proliferation, as well as decreases macrophage apoptosis in atherosclerotic plaques. Representative images and quantification of **A** Oil Red O, **B** CD68 immunostaining (scale bars 400 μm), and **C** necrotic area of aortic root sections from LXRα WT and S196A mice. Histological quantification of monocyte recruitment: **D** EdU positive Ly6C^high^ and **E** beads positive Ly6C^low^ cells normalized to the number of positive cells in the blood. **F** Ki67 (proliferation), and **G** Cleaved-Caspase 3 (apoptosis) were measured in the CD68^+^ cells. Data are expressed as mean ± SD (*n* = 13 except for ORO *n* = 10 and cleaved-Caspase 3 *n* = 7) and obtained from independent samples. *T* test; *P < 0.05, and ***P < 0.001.

The favorable lipoprotein profile exhibited by the LXRα S196A mice might lead to reduced lipid accumulation within cells of the atherosclerotic plaque. Consistent with this, we found a ~40 % reduction in the neutral lipid content within the LXRα S196A compared to WT plaque as measured by Oil Red O (ORO) staining (WT:8.82 ± 3.45, S196A:5.54 ± 1.43%, *p* = 0.0123, and WT:11908 ± 5809, S196A:7363 ± 1837 µm^2^, *p* = 0.0298; Fig. 2A and Supplementary Fig. S3A). We also measured plaque area and total macrophage content by CD68 staining. We observed trends toward a reduction in total plaque area (WT:488166 ± 143440, S196A:423317 ± 106511 µm^2^, *p* = 0.203; Supplementary Fig. S3B) and CD68^+^ cell area (WT:32.10 ± 4.45, S196A:28.72 ± 5.66%, *p* = 0.0976, and WT:156675 ± 53477, S196A:125424 ± 49816 µm^2^, *p* = 0.1362; Fig. 2B and Supplementary Fig. S3C), whereas necrotic area was unchanged (WT:146763 ± 74249, S196A:147001 ± 50371 µm^2^, *p* = 0.9925; Fig. 2C). Since plaque macrophage content reflects multiple kinetic factors including monocyte recruitment, macrophage proliferation, and apoptosis, we also measured each of these parameters. To determine the Ly6C^high^ monocytes (precursors of pro-inflammatory M1 macrophages in progressing plaques) and Ly6C^low^ monocytes (potential precursors of anti-inflammatory M2 macrophages) content, mice were injected with EdU (5-ethynyl-2’-deoxyuridine) and fluorescent latex beads, 3 days or 1 day before harvest, respectively^26^. EdU is incorporated into the DNA of proliferating Ly6C^high^ monocytes while fluorescent beads are engulfed by Ly6C^low^ monocytes. Quantification of EdU- or bead-labeled monocytes infiltrating into the plaques revealed a significant 50% decrease in the recruitment of inflammation-prone Ly6C^high^ monocytes into the plaques of LXRα S196A relative to LXRα WT mice (WT:45.09 ± 17.50, S196A:21.03 ± 13.03 EdU^+^ cells/section normalized by blood level, *p* = 0.0006; Fig. 2D), despite no changes in circulating monocyte number (Supplementary Fig. S4A–D). In contrast, we did not observe a change in the recruitment of Ly6C^low^ monocytes into the plaque between LXRα S196A and WT mice (WT: 28.10 ± 29.97, S196A:33.68 ± 36.72 beads^+^ cells/section normalized by blood level; Fig. 2E).

We next determined whether the reduction of Ly6C^high^ monocytes in the plaque of LXRα S196A mice reflected an innate feature of their movement to the site of inflammation or was an acquired property as a result of decreased plasma LDL-C. To test this we challenged LXRα S196A and WT mice intraperitoneally with the inflammatory stimulus zymosan A^27^ in normolipidemic conditions, and measured the recruitment of monocytes and neutrophils to the peritoneal cavity by flow cytometry at 4, 24, and 48 h. We found a reduction in the total number of monocytes LXRα S196A compared to WT mice at 24 h and 48 h (24 h WT:22.23 ± 5.83, S196A:11.03 ± 2.42, *p* = 0.0372; 48 h WT:40.9 ± 6.95, S196A:26.60 ± 2.52% of total CD45^+^ cells, *p* = 0.0285; Supplementary Fig. S4E). The number of neutrophils at 4 h was also reduced in LXRα S196A mice relative to WT mice (WT:74.37 ± 21.88, S196A:29.9 ± 10.88% of total CD45^+^ cells, *p* = 0.0344; Supplementary Fig. S4F). These findings indicate that LXRα S196A mice have reduced leukocyte recruitment upon inflammation suggesting that the reduction of plaque monocytes in LXRα S196A is cell autonomous and a result of decreased cell movement to the site of inflammation.

Smooth muscle cells in the plaque under high cholesterol can transdifferentiate into macrophage-like CD68^+^ cells^28^, and potentially contribute to the CD68 content. Therefore, we measured the number of cells expressing alpha smooth muscle actin (SMaA), a marker of smooth muscle cells in LXRα S196A and WT plaques and no significant difference was observed (WT:11.47 ± 9.19, S196A:14.44 ± 5.60 SMaA^+^ cells/section, *p* = 0.394; Supplementary Fig. 3D).

We observed a ~50% decrease in macrophage proliferation within the plaque in LXRα S196A relative to WT by co-staining with the proliferation marker Ki67 and the macrophage marker CD68 (WT:37.07 ± 9.80, S196A:19.48 ± 3.09 CD68^+^ Ki67^+^ cells/section, *p* < 0.0001; Fig. 2F). Similarly, we observed a ~40% reduction in apoptosis in LXRα S196A compared to WT as measured by cleaved-caspase 3 and CD68 co-staining (WT:25.39 ± 3.83, S196A:14.37 ± 3.91 CD68^+^ Cleaved-Caspase 3^+^ cells/section, *p* = 0.0002; Fig. 2G). This would result in macrophage accumulation in the plaque, and could explain why we do not observe a significant reduction in macrophage content in the plaque despite a reduction in both Ly6C^high^ monocyte recruitment and macrophage proliferation in S196A plaques.

### LXRα S196A decreases the number of M1-like macrophages in the plaque without affecting the number of M2-like macrophages, DCs, T cell subsets, and B cells

To investigate the cadre of immune cells in the plaque, we performed flow cytometry on the digested aortas from plaques of *Ldlr*^*−/−*^ mice receiving bone marrow from either LXRα S196A or WT mice (see Supplementary Fig. S5 for gating strategy). There was a significant reduction in M1 macrophages (F4/80^+^, Cd11b^+^, Cd11c^+^) (WT:7.4 ± 0.8, S196A:5.6 ± 1.3% of CD45^+^F4/80^+^ cells, *p* = 0.0269) in LXRα S196A mice compared to WT mice (Supplementary Fig. S6A, B). There was no change in the number of immune cells (CD45^+^), macrophages (CD45^+^, F4/80^+^), M2-like macrophages (F4/80^+^, Cd11b^+^, Cd11c^−^), dendritic cells (DC, CD45^+^, F4/80^−^, Cd11c^+^, IA/IE^+^), B cells (CD45^+^, F4/80^−^, B220^+^), T cells (CD45^+^, F4/80^+^, CD3^+^), and T cell subsets, including CD4, CD8, T helper and T regulatory cells (Supplementary Fig. S6A). We also found that M2-like macrophage subtype represented the largest fraction of the CD45^+^ cells in the plaque (WT:78.4, S196A:81.7%, Supplementary Fig. S6B). The fact that LXRα S196A reduces M1-like macrophages, but has no effect of the number of M2-like macrophages may also help to explain why there is only a trend toward reduced CD68^+^ staining in the plaque. Thus, sorting of immune cells from the plaque imply less accumulation of inflammatory macrophages in LXRα S196A compare to WT mice, and is consistent with the bead labeling studies.

### LXRα S196A promotes expression of genes regulating mitochondrial activity and decreases inflammatory pathways in plaque CD68^+^ cells

**Fig. 3:**
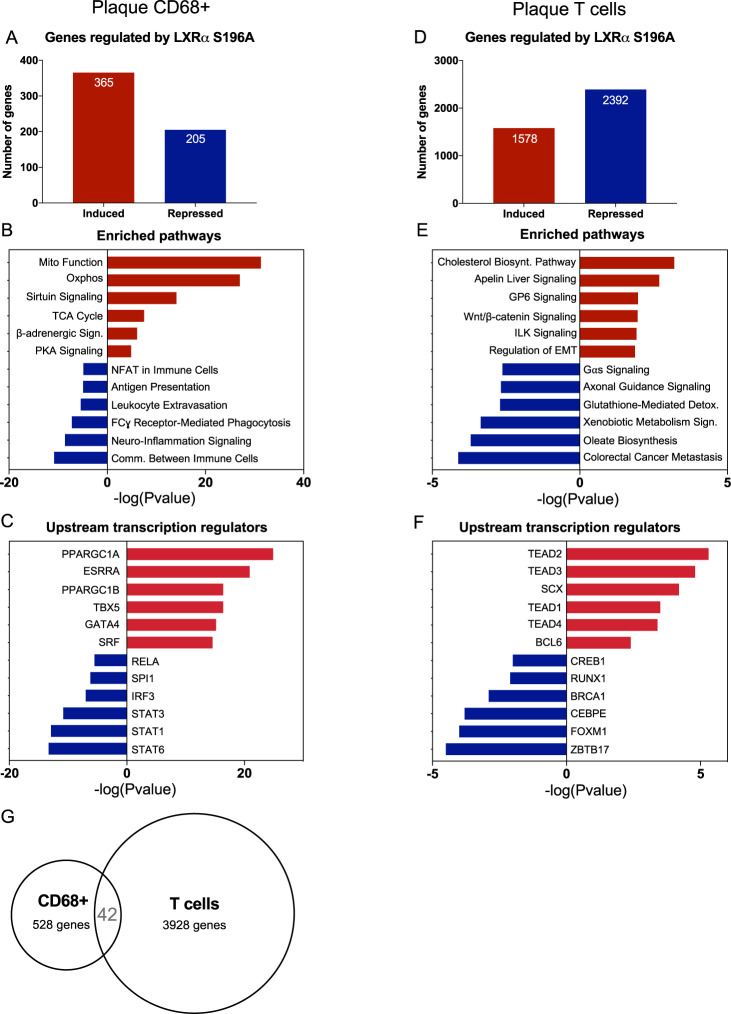
LXRα S196A promotes expression of genes regulating mitochondrial activity, decreases inflammatory pathways in plaque macrophages, and reprograms plaque T cell transcriptome. **A** Number of genes regulated by LXRα S196A versus WT by RNA seq from plaque laser captured microdissected CD68^+^ cells (LogFC > 0.6, p-value < 0.05). **B** Enriched pathways in plaque CD68^+^ in S196A after differential gene expression (DGE) analysis using Ingenuity Pathway Analysis (IPA). **C** Transcriptional regulators associate with upregulated or downregulated genes in plaque CD68^+^ cells were determined by IPA transcription factor analysis. **D** Number of genes regulated by LXRα S196A versus WT by RNA seq from sorted T cells (LogFC > 1, FDR < 0.05). **E** Enriched pathways in plaque T cells in S196A after DGE analysis using IPA. **F** Transcriptional regulators controlling the upregulated or downregulated genes in plaque T cells. **G** Comparison of the differentially expressed genes in plaque CD68^+^ versus plaque T cells.

To further elucidate the mechanisms governing macrophage inflammatory response in atherosclerosis, we examined gene expression changes from LXRα WT and LXRα S196A plaque CD68^+^ cells collected by laser-capture microdissection by performing bulk RNA sequencing (RNA seq)^29^ (Supplementary Fig. S7A and Supplementary Data 2). There was comparable expression of LXRα and LXRα S196A in plaque CD68 + cells (Table 1). We found in CD68^+^ that LXRα S196A induced 365 and repressed 205 genes compared to LXRα WT (LogFC > 0.6, *p* value < 0.05) (Fig. 3A). Ingenuity Pathway Analysis (IPA)^30^ of the genes upregulated in LXRα S196A in CD68^+^ cells identified mitochondrial function and oxidative phosphorylation (Oxphos) as the top enriched pathways, followed by sirtuin signaling, the TCA cycle, PKA signaling, and β-adrenergic signaling (Fig. 3B, Supplementary Fig. S7B and Supplementary Table S1). This suggests that mitochondrial activity and energy metabolism, characteristics of metabolic pathways upregulated in anti-inflammatory macrophages, are induced within the LXRα S196A CD68^+^ cells relative to WT^31,32^. Genes downregulated by S196A in CD68^+^ cells were associated with pro-inflammatory pathways including communication between immune cells, FCγ receptor-mediated phagocytosis, leukocyte extravasation, antigen presentation (Fig. 3B). Transcription factor analysis via IPA revealed that the majority of the genes upregulated in LXRα S196A compared to WT CD68^+^ cells are associated with PPARGC1A (PGC1α) (Fig. 3C). PGC1α is a co-activator of LXRα^33^ and a regulator of mitochondrial biogenesis and function^34,35^. PGC1α is activated by deacetylation through sirtuins^36^, and this may be strengthened through increased sirtuin signaling identified by IPA in LXRα S196A. Genes downregulated by LXRα S196A compared to WT are enriched for members of the signal transducer and activator of transcription family (STAT6, STAT1, STAT3), as well as IRF3, SPI1, and RELA (Fig. 3C). These transcription factors are activated in macrophages by inflammatory signals^37,38^, and suggest that these pathways are dampened in CD68^+^ cells expressing LXRα S196A. Thus, CD68^+^ cells within the plaque expressing LXRα S196A exhibited an increase in the expression of genes involved in mitochondrial metabolic activity and decrease in the expression of genes associated with inflammation, consistent with a less inflammatory macrophage phenotype.

**Table 1:** LXRα (WT and S196A) and LXRβ expression in CD68 + and T cells from the plaque, and FB, FBC, and T cells from the perigonadal white adipose tissue (pWAT).

GENE_SYMBOL	Plaque CD68 + WT	Plaque CD68 + S196A	Plaque T cells WT	Plaque T cells S196A	FB pWAT WT	FB pWAT S196A	FBC pWAT WT	FBC pWAT S196A	T cells pWAT WT	T cells pWAT S196A
LXRα (*Nr1h3*)	45.5	32.9	2.9	6.0	4.0	1.3	8.0	7.9	4.1	1.2
LXRβ (*Nr1h2*)	82.3	45.6	30.6	14.9	23.9	17.1	21.2	17.9	34.9	34.8

Expression of LXRα and LXRβ among different immune cell populations in plaque and pWAT as measured in transcripts per million (TPM) by RNA seq.

### LXRα S196A reprograms the T cell transcriptome in atherosclerotic plaques

It has been increasingly recognized that T cells play important roles in the pathogenesis of atherosclerosis^39^. To begin to understand the potential contribution of LXRα S196A in T cell gene expression during atherosclerosis, we performed bulk RNA seq from plaque T cells (CD45^+^F4/80^-^CD3^+^) sorted by flow cytometry (Supplementary Fig. S7C and Supplementary Data 3). There was slightly higher expression of LXRα S196A compared to LXRα WT in plaque T cells, and greater LXRβ expression compared to LXRα, which is consistent with previous studies^40^ (Table 1). We found that LXRα S196A induced 1578 genes and repressed 2,392 genes compared to LXRα WT (LogFC > 1, FDR < 0.05) (Fig. 3D). IPA of the genes induced by LXRα S196A in plaque T cells identified cholesterol biosynthesis, which is crucial for efficient T cell activation and T reg differentiation^41^, apelin signaling, which has been shown to attenuate atherosclerosis mediated by angiotensin II^42^, glycoprotein VI, which impacts platelet function^43^, Wnt/β-catenin, which play key roles in T cell differentiation, polarization and survival^44^, and Integrin-linked kinase (ILK) signaling, which has been reported to promote T-cell trafficking and survival^45^. Genes downregulated by S196A were associated with oleate biosynthesis, which supplies inflammatory fatty acids, xenobiotic metabolism, which provides ligands for PXR and inhibits T lymphocyte proliferation and anergy^46^, glutathione-mediated detoxification, which can perturb redox homeostasis and promote reductive stress and cardiovascular complications^47^ (Fig. 3E, Supplementary Table S2). Downregulation of these pathways would be predicted to reduce the synthesis of and signaling by inflammatory mediators, and restore redox homeostasis. Transcription factor analysis via IPA found that the genes upregulated in LXRα S196A are predominantly associated with the TEAD family of transcription factors (Fig. 3F). Activation by these factors has been shown to promote T reg differentiation and suppression of inflammation^48^.

Comparison of the differentially expressed genes between LXRα S196A and WT from plaque CD68^+^ and T cells showed largely non-overlapping transcriptomes, with only 42 genes shared between both types of cells (Fig. 3G). This is not surprising given the differences in lineage determining transcription factors between macrophages and T cells that control access of LXRα to its targets^49^. Thus, LXRα S196A promotes the reprograming of gene expression in both CD68^+^ and T cells within the plaque toward a less inflammatory phenotype. This likely contributes to the anti-inflammatory phenotype observed in the plaque by LXRα S196A.

### LXRα S196A increases mitochondrial respiration, ATP production and mitochondrial biogenesis in M1 and M2 BMDMs

**Fig. 4:**
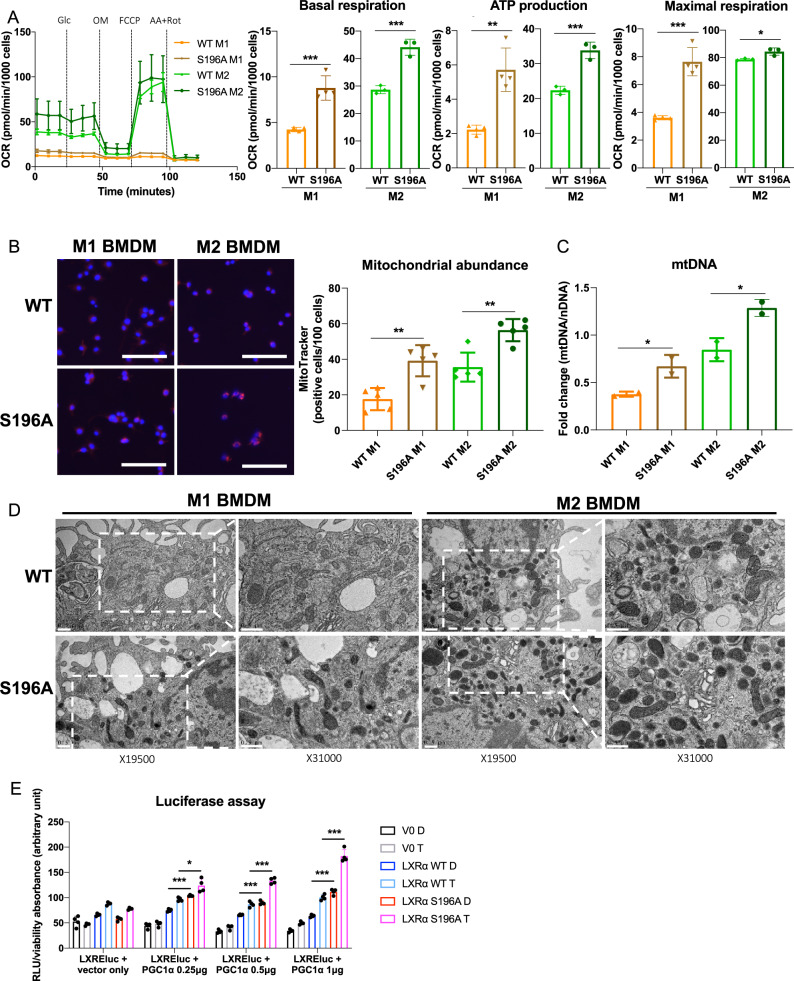
LXRα S196A BMDMs increase mitochondrial abundance and activity. Oxygen consumption rate (OCR) after sequential injection of glucose (Glc), oligomycin (OM), carbonyl cyanide 4-trifluoromethoxyphenyl hydrazine (FCCP), and antimycin plus rotenone (AA + Rot) in LXRα S196A versus WT BMDMs polarized to M1 and M2. OXPHOS parameters: basal respiration, ATP production and maximal respiration, derived from OCR values. **B** Cell imaging of mitochondrial abundance was visualized with MitoTracker Red CMXRos and quantified (scale bars: 100 µm). **C** Mitochondrial abundance in LXRα WT and S196A BMDMs determined by the expression of mitochondrial (mt) DNA normalized to nuclear (n) DNA. **D** Transmission electron micrographs of LXRα S196A versus WT BMDMs polarized to M1 and M2 (scale bars: 0.5 µm). Magnification at 19,500× and 31,000× is shown. **E** HEK293 cells stably expressing LXRα WT or LXRα S196A were transfected for 48 h with an LXRE-luciferase reporter, with either the 0.5 μg pCDNA3 vector (vector only) or with 0.25, 0.5, or 1 μg pCDNA3-PGC1α, and treated for 24 h with DMSO (D) or 5 μM T0901317 (T). Data are expressed as mean ± SD (*n* = 3/4 for OCR measurements, *n* = 5 for MitoTracker quantification, *n* = 2 for mtDNA measure and TEM, and *n* = 4 for luciferase assay,). T test; *P < 0.05, **P < 0.01, and ***P < 0.001.

Given the relationship of LXRα S196A to altered expression of genes in plaque CD68^+^ cells involved in mitochondrial oxidative phosphorylation, we assessed whether LXRα S196A enhanced mitochondrial respiration. In bone marrow-derived macrophages (BMDM) derived from LXRα WT and LXRα S196A, we determined the effect of LXRα phosphorylation on mitochondrial function by Seahorse XF Extracellular Flux analysis of the oxygen consumption rate (OCR) under pro- and anti-inflammatory conditions. In M1 and M2 BMDMs LXRα S196A showed higher basal respiration (M1 WT:4.24 ± 0.24, S196A:8.78 ± 1.33, *p* = 0.0005; M2 WT:28.70 ± 1.46, S196A:44.13 ± 2.93 pmol/min/1000 cells, *p* = 0.0012), ATP production (M1 WT:2.23 ± 0.26, S196A:5.72 ± 1.27, *p* = 0.0017; M2 WT:22.39 ± 1.17, S196A:33.81 ± 2.37 pmol/min/1000 cells, *p* = 0.0017) and maximal respiration rate (M1 WT:3.61 ± 0.15, S196A:7.64 ± 1.01, *p* = 0.0002; M2 WT:78.81 ± 0.77, S196A: 84.46 ± 2.66 pmol/min/1000 cells, *p* = 0.0242) compared to control cells (Fig. 4A), suggesting that reduction of LXRα phosphorylation promotes electron transport chain capacity in the mitochondria.

One possibility for this could be by increasing mitochondria biogenesis in LXRα S196A cells. To examine this, we quantified mitochondrial abundance in LXRα WT and S196A BMDMs by labeling the mitochondria within live cells using the mitochondrial-specific probe, MitoTracker^50^. Consistent with the enhanced mitochondrial activity in LXRα S196A, we found increased MitoTracker positive cells (M1 WT:17.60 ± 5.60, S196A:39.20 ± 7.90, *p* = 0.001; M2 WT:35.60 ± 7.30, S196A: 56.40 ± 5.60 positive cells/100 cells, *p* = 0.0022; Fig. 4B) and an increase in mitochondrial (mt) DNA (M1 WT:0.38 ± 0.27, S196A:0.67 ± 0.12, *p* = 0.047, M2 WT:0.85 ± 0.12, S196A:1.20 ± 0.09 fold change, *p* = 0.035; Fig. 4C) in M1 and M2 LXRα S196A cells compared to LXRα WT.

Since LXRα S196A gene expression in plaque CD68^+^ cells converge on the mitochondrion, we evaluated mitochondrial morphology, which is crucial for the maintenance of the mitochondrial membrane potential and ATP production^51^ by performing transmission electron microscopy of LXRα WT and S196A M1 and M2 BMDMs (Fig. 4D). We observed alterations in mitochondrial morphology and ultrastructural changes in M1 LXRα WT BMDMs (less electron dense cristae) as compared with M1 LXRα S196A BMDMs (more electron dense cristae). The mitochondria observed in M1 LXRα S196A BMDMs look similar to mitochondria found in M2 BMDMs from both WT and LXRα S196A. This is consistent with the mitochondria from M1 LXRα S196A producing ATP in part through oxidative phosphorylation (Fig. 4A), a mechanism that is normally found in the mitochondria of anti-inflammatory macrophages^52^.

PGC1α has been reported to function as a coactivator for LXRα and is a more potent coactivator under conditions when cells are treated with both LXR and RXR ligands^33^, which we have reported promotes the non-phosphorylated form of LXRα^15^. This suggests that LXRα S196A could more efficiently utilize PGC1α as a coactivator than LXRα WT. To test this, we transfected into HEK293 cells stably expressing either LXRα WT or LXRα S196A with an LXR-responsive luciferase reporter gene, along with increasing amounts of PGC1α expression plasmid in the absence and presence of the LXRα ligand, T0901317. In the presence of increasing PGC1α we found a greater increase in both basal and T0901317-dependent luciferase activity in cells expressing LXRα S196A as compared LXRα WT (PGC1α 0.25 µg WT D:74.88 ± 2.67, S196A D: 103.80 ± 1.29, *p* < 0.0001, WT T:96.59 ± 3.656, S196A T: 123.92 ± 13.22, *p* = 0.0175; PGC1α 0.5 µg WT D: 65.88 ± 1.06, S196A D:90.00 ± 3.56, *p* = 0.0006, WT T:86.72 ± 5.31, S196A T:133.28 ± 5.84, *p* = 0.0002, PGC1α 1 µg WT D:63.73 ± 2.33, S195A D:11.37 ± 5.62, *p* < 0.0001, WT T:100.43 ± 5.49, S196A T:182.42 ± 12.78 RLU/viability, *p* = 0.0008; Fig. 4E). This is consistent with PGC1α coactivating LXRα S196A more efficiently than phosphorylated LXRα WT. Thus, LXRα S196A drives enhanced mitochondrial respiration and biogenesis via increased mitochondrial gene expression (Fig. 3B) likely through PGC1α (Fig. 4B, C).

### LXRα S196A protects from diet-induced obesity

**Fig. 5:**
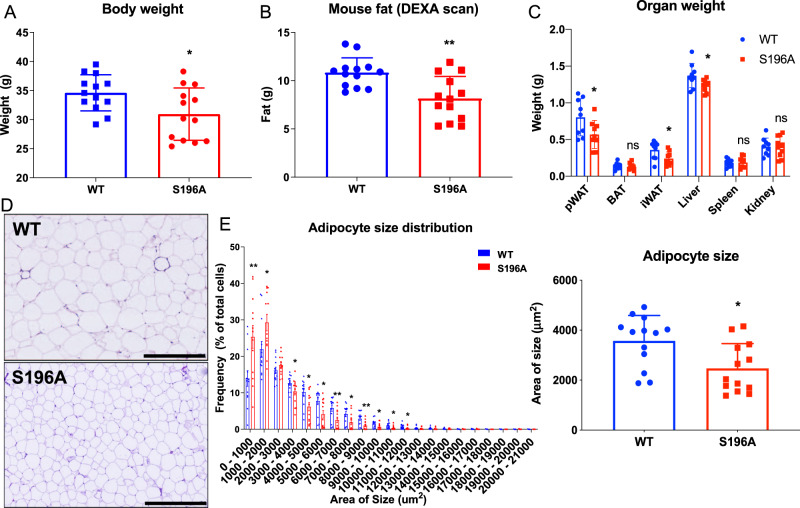
LXRα S196A reduces body weight and fat accumulation in mice. **A** Body weight of LXRα WT and S196A mice. **B** The total body fat mass was measured by DEXA scan. **C** Organs were collected and weighed: peritoneal white adipose tissue (pWAT), brown adipose tissue (BAT), inguinal white adipose tissue (iWAT), liver, spleen, and kidney. **D** Representative images of hematoxylin and eosin-stained pWAT from LXRα WT and S196A mice. **E** Quantification of the frequency of adipocytes at indicated size ranges and adipocyte size in LXRα WT and S196A mice. Scale bars: 400 µm. Data are expressed as mean ± SD (*n* = 13 per group except for organs weight *n* = 10) and obtained from independent samples. *T* test; *P < 0.05 and **P < 0.01.

Obesity and atherosclerosis share pathological features including lipid accumulation, inflammation, and cytokine production mediated by both innate and adaptive immune cells that accumulate within their respective tissues^10,53–55^. We therefore examined whether LXRα S196A affected fat deposition and immune cell recruitment in adipose tissue in the same mice as those analyzed for atherosclerosis progression. LXRα S196A mice showed less weight gain during the experiment compared to WT mice (Supplementary Fig. S8A). Mice reconstituted with LXRα S196A bone marrow weighed 15% less than their WT counterparts after 16 weeks of western diet feeding (WT:34.6 ± 3.1, S196A:31.0 ± 4.5 g, *p* = 0.0236; Fig. 5A), despite equivalent food intake during the course of the experiment (Supplementary Fig. S8B). This was observed in three independent cohorts. We confirmed that total body fat mass was lower in LXRα S196A compared to control WT mice by Dual energy X-ray absorptiometry (DEXA) scanning (WT: 10.8 ± 1.5, S196A:8.2 ± 2.3 g, *p* = 0.0018; Fig. 5B). Slight differences were observed in bone mineral density, but no differences were found in lean body mass between LXRα S196A and LXRα WT mice (bone mineral density WT:0.059 ± 0.002, S196A:0.056 ± 0.003 g/cm^3^, *p* = 0.0210; lean mass WT:23.75 ± 1.61, S196A:23.15 ± 1.75 g, *p* = 0.3718; Supplementary Fig. S8C). LXRα S196A mice relative to WT also showed reduced perigonadal white adipose tissue (pWAT) and inguinal white adipose tissue (iWAT) weight, as well as reduced liver weight (pWAT WT:0.80 ± 0.25, S196A:0.57 ± 0.19, *p* = 0.0414; iWAT WT:0.35 ± 0.12, S196A:0.24 ± 0.09, *p* = 0.0320; liver WT:1.37 ± 0.17, S196A:1.24 ± 0.09 g, *p* = 0.0465; Fig. 5C), without a change in brown adipose tissue (BAT) weight. The change in VAT is associated with a decrease in adipocyte size in LXRα S196A compared to WT mice (WT:3570 ± 1018, S196A:2471 ± 990.1 µm^2^, *p* = 0.0102; Fig. 5D-E), consistent with less lipid accumulation. The number of macrophages determined by F4/80^+^ cell staining was reduced in S196A compared to WT pWAT (WT:32.7 ± 1.7, S196A:30.1 ± 3.6%, *p* = 0.0372; Supplementary Fig. S8D, E). A trend toward decreased numbers of crown-like structures, a hallmark of the pro-inflammatory processes in adipose tissue^11,56^, was also observed (WT:0.55 ± 0.66, S196A:0.15 ± 0.17 number of crown-like structure per field, *p* = 0.0549; Supplementary Fig. S8F).

A possibility for the adipocyte phenotype is that the myeloid cells from the LXRα S196A signal to the adipocytes and decrease the expression of genes involved in lipid accumulation or increase the expression of genes involved in lipolysis. We therefore measured from the pWAT adipocytes of mice reconstituted with bone marrow from either LXRα WT or S196A the expression of *Fabp4* (Fatty acid binding protein 4), which promotes fatty acid uptake, and *Plin2* (Perilipin2), which coat intracellular lipid droplets to develop and maintain adipose tissue. We also examined the expression in adipocytes of lipolysis activators *Atgl* (Adipose triglyceride lipase) and *Hsl* (Hormone-sensitive lipase), which hydrolyze stored triglycerides in adipose tissue. We did not observe any changes in expression of these genes in pWAT adipocyte from either LXRα S196A or WT mice (*Fapb4* WT:2.26 ± 0.69, S196A:1.78 ± 0.42, *p* = 0,1665; *Plin2* WT:3.95 ± 2.97, S196A:2.13 ± 1.20, *p* = 0.1921; *Atgl* WT:2.40 ± 1.15, S196A:1.97 ± 1.52, *p* = 0.59; *Hsl* WT:3.71 ± 2.04, S196A:2.88 ± 1.00 fold change, *p* = 0.3917; Supplementary Fig. S9A, B). We also determined by western blot from pWAT the protein abundance of FABP4, ATGL, HSL, phospho-HSL, which is associated with HSL activation, as well as ADIPOQ and PLIN2 in mice reconstituted with bone marrow from LXRα S196A and WT mice. Although there was mouse to mouse variability in the protein abundance of these factors in pWAT from both genotypes, there were no significant changes in protein abundance of FABP4, ATGL, HSL, phospho-HSL, ADIPOQ, and PLIN2 in LXRα S196A versus WT adipose tissue (ADIPOQ WT:0.98 ± 1.15, S196A:1.08 ± 0.22, *p* = 0.4194; FABP4 WT:0.98 ± 0.43, S196A:0.66 ± 0.22, *p* = 0.1782; PLIN2 WT:1.00 ± 0.67, S196A: 1.46 ± 0.58, *p* = 0.2814; ATGL WT 1.00 ± 0.36, S196A: 1.08 ± 0.25, *p* = 0.6938; phosphor-HSL WT: 1.02 ± 0.59, S196A:1.10 ± 0.55, *p* = 0.8308; HSL WT:1.00 ± 0.25, S196A:1.14 ± 0.37 signal intensity relative to HSP90, *p* = 0.5019; Supplementary Fig. S9C). We did however find that in the plasma the level of ADIPOQ was higher in LXRα S196A compared to its wild type counterpart (WT:50931 ± 6042, S196A:74087 ± 7966 ng/mL, *p* = 0.0391; Supplementary Fig. S9D). This is in agreement with reports that the plasma levels of adiponectin are higher in lean versus obese rodent and humans^57,58^. Thus, the reduction in fat accumulation by S196A cannot be explained by changes in the expression of proteins commonly associated with lipid accumulation or lipolysis. Rather, the phenotype appears more complex, with increased adiponectin protein secretion as a potential systemic factor affecting lipid accumulation in S196A.

Given that BAT has the capacity to control energy homeostasis^59^, we also examined whether BAT morphology and UCP1 levels in LXRα S196A relative to LXRα WT mice. BAT weight was not affected in LXRα S196A (Fig. 5C). BAT tissue morphology and UCP1 protein abundance by western blot and by immunohistochemistry (IHC) were unchanged in S196A versus WT mice on western diet (Supplementary Fig. S9E, F). This suggests that BAT function is not contributing to the lower adipose deposition observed in LXRα S196A compared to WT.

Another possibility for the reduced adiposity in S196A versus WT mice could be differences in their locomotor activity and/or energy expenditure. To test this, we evaluated the effects of LXRα S196A on energy balance by performing metabolic cage studies on *Ldlr*^*−/−*^ mice reconstituted with LXRα WT and S196A on western diet, over two light-dark cycles. We found no difference in locomotor activity or energy expenditure (EE) (Supplementary Fig. S10A and B). We did observe in the S196A mice an increase in the respiratory exchange ratio (RER) during the night cycle when mice are active compared to WT mice (dark cycle WT:0.88 ± 0.0, S196A: 0.92 ± 0.01, *p* = 0.0045; light WT:0.87 ± 0.2, S196A:0.87 ± 0.2 kcal expended/hour, *p* = 0.9457; Supplementary Fig. S10C). This suggests that the fuel used for metabolism in LXRα S196A mice is more dependent on carbohydrates relative to WT mice, and consistent with the reduction in fat mass in LXRα S196A. These data demonstrate that LXRα S196A expressed in the bone marrow protects *Ldlr*^*−/−*^ mice from diet-induced obesity when challenged by a western diet in part through a greater consumption of carbohydrates, which would reduce one of the substrates for lipid synthesis in adipocytes in LXRα S196A compared to WT mice.

### LXRα S196A shows less inflammatory adipocyte tissue macrophages (ATM) and provokes a reprograming of gene expression in ATM and T cells in pWAT

**Fig. 6:**
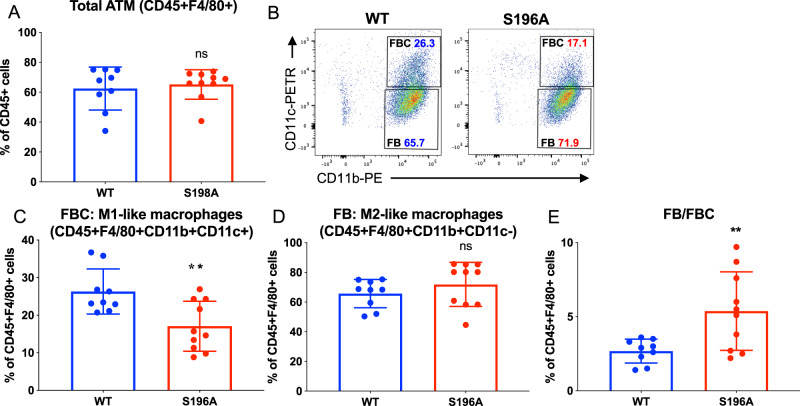
LXRα S196A ATMs are less inflammatory. Immune cells in pWAT from LXRα WT and S196A were analyzed by flow cytometry: **A** adipose tissue macrophages (ATM), **B** FBC and FB populations, **C** FBC (F4/80^+^, CD11b^+^, CD11c^+^) as a percentage of CD45^+^F4/80^+^ cells, and **D** FB (F4/80^+^, CD11b^+^, CD11c^-^) as a percentage of CD45^+^F4/80^+^ cells, and **E** ratio FBC/FB. Data are expressed as mean ± SD (*n* = 9/10 per group) and obtained from independent samples. *T* test; **P < 0.01.

Analysis of the immune cells present in the pWAT by flow cytometry revealed LXRα S196A affects the abundance a specific immune cell population (for gating strategies see Supplementary Fig. S11A). The number of total immune cells (CD45^+^ WT:62.43 ± 14.35, S196A:65.19 ± 9.86%, *p* = 0.6288. Supplementary Fig. S12A), and ATM were similar in S196A compared to WT (CD45^+^F4/80^+^ WT:62.43 ± 14.36, S196A:65.19 ± 9.86%, *p* = 0.6288; Fig. 6A). The number of pro-inflammatory FBC macrophages (M1-like: F4/80^+^CD11b^+^CD11c^+^) was reduced (WT:26.31, S196A:17.07 ± 6.64%, *p* = 0.0056; Fig. 6B, C), while the number of anti-inflammatory FB macrophages (M2-like: F4/80^+^CD11b^+^CD11c^−^) was unchanged (WT:65.89 ± 9.52, S196A:71.91 ± 14.87%, *p* = 0.2989; Fig. 6B and D). This resulted in a higher ratio of inflammation resolving FB versus inflammation promoting FBC macrophages in LXRα S196A compared to LXRα WT adipose tissue (WT:2.67 ± 0.81, S196A:5.38 ± 2.65%, *p* = 0.0093; Fig. 6E). The percentage of dendritic cells (DC), B cells, and T cell populations did not change between WT and LXRα S196A (Supplementary Fig. S12A, B). Therefore, the LXRα S196A expressing mice resulted in fewer inflammatory ATM in pWAT, characteristic of a leaner phenotype.

**Fig. 7:**
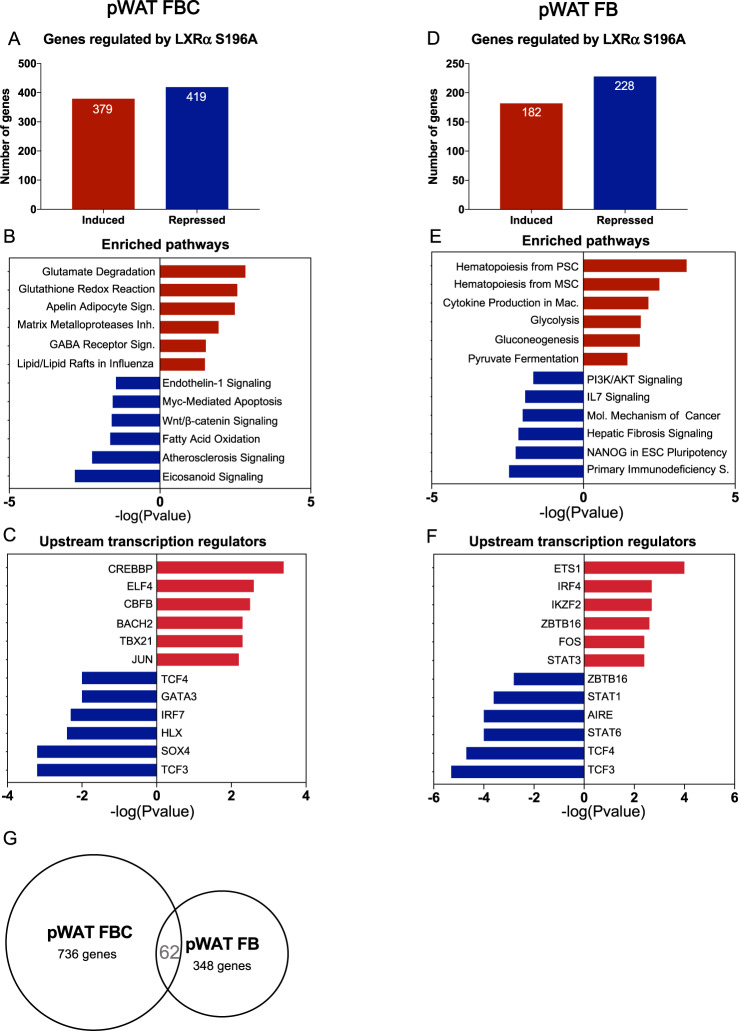
LXRα S196A ATMs regulate pathways involved in inflammation, cell death, proliferation, and metabolism. RNA seq analysis of pWAT FBC and FB from LXRα S196A vs WT mice. **A** Number of genes regulated in pWAT FBC by LXRα S196A versus WT by RNA seq (LogFC > 1, FDR < 0.05). **B** Enriched pathways in pWAT FBC in S196A after DGE analysis with IPA. **C** Transcriptional regulators controlling the upregulated or downregulated genes in pWAT FBC. **D** Number of genes regulated in pWAT FB by LXRα S196A versus WT by RNA seq (LogFC > 1, FDR < 0.05). **E** Enriched pathways in pWAT FB in S196A after DGE analysis with IPA. **F** Transcriptional regulators controlling the upregulated or downregulated genes in pWAT FB. **G** Number of common genes in pWAT FBC and FB.

To explore pathways underlying the changes observed in the ATM phenotype and in T cells, FBC, FB and T cells were sorted from the pWAT of bone marrow reconstituted with LXRα S196A and WT mice and RNA seq was performed. There was comparable expression of LXRα WT and LXRα S196A in FBC and FB cells (Table 1). Consistent with studies demonstrating lower expression of LXRα compared to LXRβ in T cells^40^, we also observed less expression of LXRα WT and LXRα S196A relative to LXRβ in T cells from the pWAT (Table 1). We analyzed genes differentially expressed in LXRα S196A vs WT in FBC (LogFC > 1, FDR < 0.05, Supplementary Fig. S13A and Supplementary Data 4). We found that LXRα S196A repressed 419 genes and induced 379 genes compared to LXRα WT (Fig. 7A). IPA analysis of upregulated genes in LXRα S196A revealed that glutamate degradation and glutathione redox are the most significant pathways associated with LXRα S196A (Fig. 7B and Supplementary Table S3). Degradation of glutamate would be predicted to skew macrophages toward an anti-inflammatory M2 phenotype. Glutathione inhibits the inflammatory response involving reactive oxygen species, and would also be expected to reduce inflammation. Transcription factor analysis via IPA revealed that the majority of the genes upregulated in LXRα S196A compared to WT FBC cells from pWAT are associated with CREBBP, ELF4, CBFB, and to a lesser extent BACH2, TBX21 and JUN. CREBBP inhibits inflammation-induced macrophage apoptosis^60^. Genes downregulated by S196A were associated with a reduction in eicosanoid signaling that would decrease the inflammatory response^61^ (Fig. 7B). TCF3 and SOX4 were the major transcription factors associated with genes in these pathway classes (Fig. 7C). Because enhanced TCF3 activity in macrophages has been shown to be associated with chronic inflammation^62^ and that increased SOX4 expression has linked to cell survival and migration^63^, a reduction in the activity of these transcriptions would be likely to reduced inflammation and migration of FBC cells in LXRα S196A vs LXRα WT, and is consistent with our findings.

We next analyzed genes differentially expressed in LXRα S196A vs WT in FB cells from pWAT (LogFC > 1, FDR < 0.05, Supplementary Fig. S13B and Supplementary Data 4). LXRα S196A induced 182 genes and repressed 228 genes (Fig. 7D). IPA analysis demonstrated that pathways involved in stem cell hematopoiesis (hematopoiesis from pluripotent stem cells and mesenchymal stem cells), cytokine production, and glucose/energy metabolism (glycolysis, gluconeogenesis, and pyruvate fermentation) were enriched in the induced genes in FB LXRα S196A (Fig. 7E). Transcription factors associated with the upregulated genes were ETS1, IRF4, IKZF2, ZBTB16, FOS, and STAT3 (Fig. 7F). IPA analysis showed that genes downregulated in pWAT FB LXRα S196A were associated with inflammation (Primary immunodeficiency, and IL7 signaling); hepatic fibrosis signaling; cells polarization, survival, and proliferation (Fig. 7E and Supplementary Table S4). TCF3, TCF4, STAT6, AIRE, STAT1, ZBTB16 were the transcription factor upstream of the downregulated genes pWAT FB LXRα S196A (Fig. 7F).

Comparison of pWAT FBC and FB RNA seq showed that only 62 genes were shared between both type of cells in pWAT (Fig. 7G). This suggests independent regulation of gene expression by LXRα S196A in inflammatory and anti-inflammatory macrophages.

We also examined T cells from pWAT by RNA seq and found 624 genes were induced and 622 genes were repressed in LXRα S196A vs WT (LogFC > 1, FDR < 0.05; Supplementary Fig. S13C, D and Supplementary Data 5). IPA analysis showed an upregulation of genes involved in T helper differentiation, activation, and function (Th1 and Th2 activation, Th1, Th2, crosstalk between dendritic cells and natural killer T cells, T helper cell differentiation, and iCOS-iCOSL signaling in T helper cells) (Supplementary Fig. S13E, and Supplementary Table 5). This suggests the control of T cells homeostasis by LXRα phosphorylation. Transcription factor analysis showed that ZBTB16, GATA3, NFATC1, IKZF2, PPARG, and TCF7 were associated with the upregulated genes in pWAT T cells RNA seq (Supplementary Fig. S13F). Genes downregulated in pWAT T cells RNA seq, were part of inflammatory processes (complement system, antigen presentation), RXR activation (LXR/RXR activation, VDR/RXR activation), phagocytosis (FCγ receptor mediated phagocytosis), and atherosclerosis signaling (Supplementary Fig. S13E). RXRA, TCL1A, TP73 STAT6, RXRB, and STAT1 were the transcription factor linked to the downregulated genes in pWAT T cells RNA seq (Supplementary Fig. S13F). Because LXR forms heterodimers with RXR and is activated by ligands of either partner^64^, this suggests a dampening of LXR signaling in this context. Together, these data suggest that reduction of pS196 in the bone marrow induces a transcriptional reprogramming of ATMs and T cells, and decreases inflammatory signaling to adipocytes. This could explain in part the decrease in lipid accumulation and in adipocyte hypertrophy in pWAT, leading to the reduction in obesity in LXRα S196A compared to WT mice.

It is noteworthy that of the cytokines from T cells with the potential for modulating macrophage activation, only *Il4* expression was increased in plaque T cells (logFC = 5.33, FDR = 0.0004) and in pWAT T cells (logFC = 3.54, FDR = 0.036) in LXRα S196A compared to WT mice (Supplementary Data 3 and 5, and Supplementary Table S6). This is consistent with IL4 promoting an immune-suppressive environment by antagonizing pathogenic Th1 and promoting Th2 responses, and in turn antagonizing M1 macrophage expansion^65^.

### Conditioned media from LXRα S196A BMDMs reduces lipid accumulation in 3T3-L1 cells

**Fig. 8:**
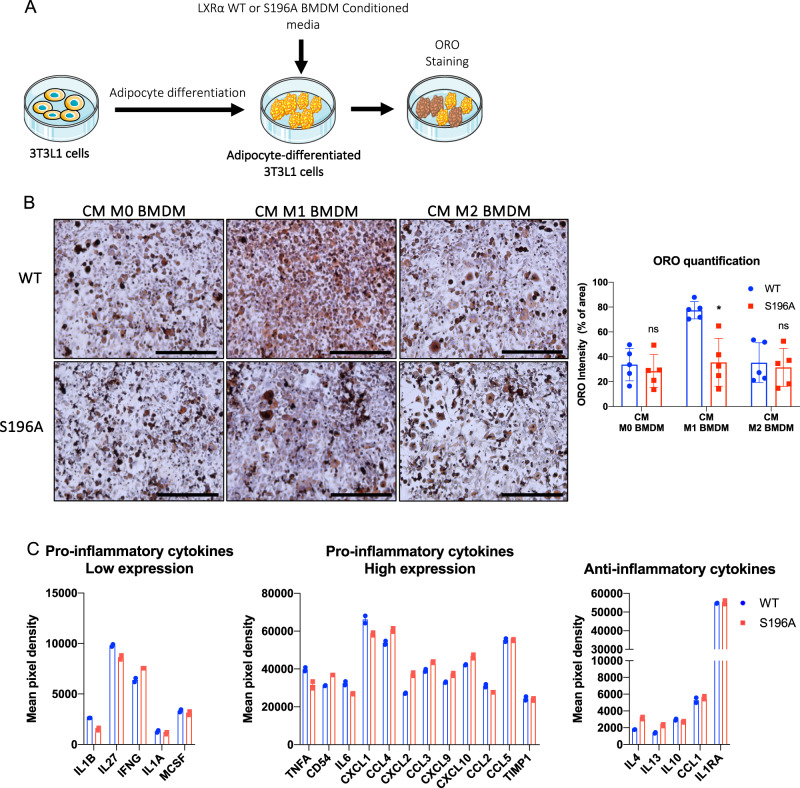
LXRα S196A BMDMs alter lipid accumulation of 3T3-L1 and is associated with the changes in secreted cytokines. **A** Experimental design: 3T3-L1 cells were differentiated into adipocytes and then treated with conditioned media from WT or S196A BMDMs (M0, M1, or M2) for 24 h and neutral lipid accumulation was determined by Oil Red O (ORO) staining. **B** Shown are representative images and quantification, of ORO staining of differentiated 3T3-L1 adipocytes treated with M0, M1 or M2 conditioned media from LXRα WT or S196A BMDMs. **C** Pro-inflammatory (low and high expression) and anti-inflammatory cytokines profiles of M1 WT and S196A BMDM. Scale bars: 400 µm. Data are expressed as mean ± SD (*n* = 5) and obtained from five independent experiments for ORO quantification. *T* test; *P < 0.05. Cytokines profiles were obtained from two independent experiments. Error bars represent the deviation from the mean.

Our previous results strongly suggest that signals from immune cells (most likely ATMs) to adipocytes are capable of reducing lipid accumulation in pWAT in mice reconstituted with LXRα S196A compared to WT (Fig. 5C–E). To test this, we developed an in vitro assay whereby 3T3-L1 cells are differentiated into adipocytes, treated for 24 h with filtered conditioned media (CM) from either LXRα WT or S196A BMDMs under different inflammatory states (M0, M1, or M2), and stained for neutral lipids by ORO to determine lipid accumulation (Fig. 8A). We found that conditioned media from M1 WT BMDMs promoted more lipid accumulation in adipocytes than conditioned media from M1 S196A BMDMs, whereas conditioned media from M0 and M2 from either WT or S196A BMDMs showed no differences in lipid accumulation (CM M0 BMDM WT:33.74 ± 13.03, S196A:28.49 ± 13.35, *p* = 0.5466; CM M1 BMDM WT:77.42 ± 6.96, S196A: 35.56 ± 19.22, *p* = 0.0018; CM M2 BMDM WT:35.28 ± 16.06, S196A:31.43 ± 1534% ORO intensity, *p* = 0.7084; Fig. 8B). This suggests M1 S196A cells secrete differing factors than M1 WT macrophages; a decrease in pro-inflammatory and/or an increase in anti-inflammatory factors, that promotes lipid accumulation.

We, therefore, compared the level of cytokines in the media of M1 LXRα WT and S196A BMDMs using a cytokine array. We found in S196A relative to WT conditioned media a decrease in the pro-inflammatory cytokines IL1β, IL27, and TNFA, IL6, and CXCL1, as well as an increase in IFNG (Fig. 8C). The anti-inflammatory cytokine IL4, and IL13 were increased in S196A conditioned media compared to WT BMDMs (Fig. 8C). Our findings suggest that LXRα S196A M1 macrophages suppress lipid accumulation in adipocytes by reducing and/or enhancing the levels of pro-inflammatory and anti-inflammatory cytokines.

## Discussion

We examined the effects of LXRα pS196 on atherosclerosis progression and obesity within the same mouse by expressing LXRα S196A in a bone marrow transplant model in *Ldlr*^−/−^ recipients. We found that expression of LXRα S196A increased HDL-C and reduced LDL-C levels. Consistent with this is a decrease in neutral lipid accumulation in plaque macrophages expressing S196A. The favorable lipoprotein profile in S196A mice is likely through a combination of increased numbers of KC recruited from the periphery to the liver in the context of bone marrow transplantation after whole body irradiation to increase LDL-C uptake^66–68^, as well as increased TG export from the liver, and increased clearance of TG from the intestine.

We also found that expression of LXRα S196A reduced the recruitment to the plaque of inflammation-prone Ly6C^high^ monocytes by both bead labeling and flow cytometry. This in turn would reduce these monocytes becoming macrophages, thus blunting atherosclerotic plaque formation, although this reduction would be opposed by decreased macrophage apoptosis. The reduction on monocyte recruitment to a site of inflammation in S196A compared to WT mice appears cell intrinsic rather than a result of changes in lipoprotein status (Supplementary Fig. 4E, F).

We also observed from the CD68^+^ cells laser captured from the plaque of LXRα S196A mice an anti-inflammatory gene expression signature associated with the upregulation of genes involved in oxidative phosphorylation, and mitochondrial activity (Fig. 3B). PGC1α, a transcriptional coactivator that coordinates mitochondrial biogenesis^34,35,69^ and an LXRα coactivator^33^, was the top putative transcriptional regulator in plaque CD68^+^ cells expressing LXRα S196A. PCG1α is activated by deacetylation via sirtuins^36^, and one of the top IPA pathways induced by LXRα S196A in CD68^+^ cells is sirtuin signaling. This suggests that the non-phosphorylated form of LXRα at S196 in a feed forward loop helps activate PGC1α via deacetylation and preferentially utilize PGC1α as a coactivator. This is consistent with the degree of transcriptional coactivation by PGC1α being greater in cells expressing LXRα S196A compared LXRα WT.

Consistent with changes in genes linked to mitochondrial activity, we demonstrated in S196A BMDMs polarized in vitro to the M1 or M2 states, upregulation of basal respiration, ATP production (Fig. 4A), increased mitochondrial abundance (Fig. 4B, C), and mitochondria morphology reminiscent of M2 BMDMs (Fig. 4D). These findings are consistent with S196A expression in CD68^+^ cells in the plaque acquiring the characteristics of anti-inflammatory macrophages^31^. Preliminary ChIP-seq studies in BMDMs suggests that LXRα occupies a majority of genes involved in mitochondria respiration, including *Atp5o, Atp5g1, Ndufb9, Ndufa5, Cycs, Cox6a2, Uqcrfs1, Ndufa4, Cox6c, Cox8b* (*unpublished observation*), suggesting direct regulation of these genes by LXRα.

We also found that expression of LXRα S196A in the bone marrow significantly reduced mouse weight, total fat, pWAT, and adipocyte size in pWAT compared to WT (Fig. 5). This phenotype was not reported in macrophage-specific S196A expressing mice in the *Ldlr*^−/−^ background^17^, suggesting that other bone marrow-derived cells in combination with macrophages are promoting this phenotype. This was not as a result of changes in BAT magnitude or BAT UCP1 expression, or alterations in activity or energy expenditure. Nor did we find expression of UCP1 in the pWAT suggesting the that the effect of S196A on pWAT was not a result of conversion into beige fat via UCP1^70^. The decrease in adipocyte size in LXRα S196A was associated with reduced numbers of pro-inflammatory ATMs in the adipose tissue (Fig. 6A–E), and a large change in the LXRα S196A transcriptomes between ATM subtypes that indicated a less inflammatory phenotype.

So why do the LXRα S196A mice gain less weight and have smaller adipocytes than their WT counterparts on a western diet despite similar food intake and energy expenditure? One option is the observed decreased in lipid absorption in the intestine of LXRα S196A compared to WT mice (Supplementary Fig. S2C), which is to decrease the entry of lipids into adipocytes. Another possibility is that because the LXRα S196A mice have higher RER when active (Supplementary Fig. S10C), they use more carbohydrate as their fuel source than WT mice, resulting in less carbohydrate being converted to stored lipids. This greater use of carbohydrate for fuel is consistent with a decrease in steady state blood glucose levels in LXRα S196A compared to WT mice without a change in insulin or glucagon levels (Supplementary Fig. S14). In addition, given that LDL also provides substrate for lipid storage in mouse adipocytes through LDLR-independent mechanisms^71–74^, and that the LXRα S196A mice have lower LDL levels, this might also contribute to the reductions in weight and adipocyte size. Thus, it is plausible that as a result of a combination of decreased lipid absorption, lower glucose and LDL levels in LXRα S196A mice, there is less lipid accumulation in the adipocytes compared to WT mice.

Turning to the reduction in lipids when conditioned media from LXRα S196A M1 BMDMs are placed onto 3T3-L1 adipocytes, it is unlikely to be the result of changes in the expression of enzymes associated with lipid accumulation or breakdown, which is similar to what we observed in vivo. We suggest by analogy to our in vivo findings that the decrease in lipid accumulation in adipocytes in vitro in part results from a change in the abundance or composition of the lipids secreted into the media from the WT and S196A BMDMs and entering the adipocytes as substrate for lipogenesis. Changes in cytokine levels or other factors could also modulate substrate availability, the activities of lipid synthesis or catabolism enzymes (versus their expressions, which were not altered). While beyond the scope of this study, as an initial approach, determining the level and composition of the lipids secreted from WT and S196A BMDMs using lipidomics could shed light on the mechanism governing lipid accumulation in adipocytes.

Our findings complement an elegant study by Gage et al. that demonstrated a myeloid-specific knock-in of LXRα S196A using LysM-cre resulted in greater macrophage proliferation via the LXRα S196A-dependent upregulation of FOXM1, and increased atherosclerosis^17^. The difference in the phenotypes between the myeloid-specific expression of S196A versus our bone marrow transplant model, in which the entire innate and adaptive immune cell compartments express LXRα S196A, suggests that LXRα S196A expression within additional bone marrow-derived cells restrains macrophage proliferation in the plaque to deter atherosclerosis. In support of this idea, we find *Il4* as being induced in T cells from plaques (and pWAT) in S196A compared to WT mice (Supplementary Data 3 and 5, and Supplementary Table S6). Both LXRα WT and S196A are expressed in T cells albeit lower compared to LXRβ, consistent with previous studies^40^, we suggest that LXRα S196A is driving the gene expression changes in T cells, although we cannot rule out cross talk from myeloid cells or alterations in circulating lipid levels. IL4 can antagonize M1 expansion and polarization, while promoting an inflammation resolving M2 phenotype, and is consistent with the reduction in inflammatory macrophage numbers in plaque CD68^+^ cells and ATMs from S196A relative to WT mice. Since in the monocyte-specific LXRα S196A mouse the T cells would be expressing LXRα WT, and the increase in for example *Il4* would not occur, it suggests a mechanism for the phenotypic differences between myeloid-specific and bone marrow transplant models (Supplementary Fig. S15).

In summary, we showed that reducing LXRα pS196 in bone marrow cells of *Ldlr*^*−/−*^ mice reduced VAT weight and attenuated atherosclerosis. This was accompanied by reduction in macrophage recruitment to the site of inflammation, and a shift in macrophages and T cells to a less inflammatory state in LXRα S196A compared to WT in both tissue microenvironments through reprograming of LXRα-dependent transcription.

## Methods

### Animals

C57BL/6J LXRα S196A mice were generated by Ozgene, and have been described^17,20^. The mice were cared for in accordance with the National Institutes of Health guidelines and the NYU Institutional Animal Care and Use Committee. Bone marrow from male LXRα WT or LXRα S196A were transplanted into 10 weeks old male *Ldlr*^*−/−*^ mice (Jackson Laboratory; Stock No: 002207). After 4 weeks after the bone marrow transplant, the mice were placed on a western diet (42% kcal fat, 0.3% kcal cholesterol, Research Diets) for 16 weeks to allow development of moderately advanced atherosclerotic plaques. To study monocyte recruitment, EdU (1 mg/30 g mouse weight) was injected intraperitoneally (IP) 3 days before harvest to label Ly6C^high^ monocytes, and circulating Ly6C^low^ monocytes were labeled by injecting mice in the retro-orbital vein with 250 μL of 1μm Fluoresbrite fluorescein isothiocyanate-dyed (YG) plain microspheres (Polysciences Inc.) diluted in PBS (1:4) 1 day before harvesting as described^26^. Mice were weighed once a week and on harvest day. Mice were anesthetized with xylazine/ketamine, and blood was collected via cardiac puncture for plasma analyses. Mice were perfused with 10% sucrose/saline. Organs weight was measured. Aortic roots were dissected and digested for further flow cytometry analysis, or embedded in optimal cutting temperature (OCT) compound medium and frozen immediately, and stored at −80 °C until further use. Liver, pWAT, and BAT were collected, frozen and stored at −80 °C, or fixed in formalin and then embedded in paraffin for further IHC studies. pWAT was also collected, digested for immune cells analysis, and adipocytes were collected in Trizol (Invitrogen). The data presented are from male mice to focus on a single experimental cohort with similar body size and hormonal milieu.

### Mouse genotyping

Genotyping was performed by mouse tail DNA PCR analysis. Genotyping was performed using the following PCR primers: R2: 5′-AAGCATGACCTGCACACAAG-3′; WT: 5′-GGTGTCCCCAAGGGTGTCCT-3′; and SA: 5′-GGTGTCCCCAAGGGTGTCCG-3′. Primers R2 and WT identify the wild-type allele (642-bp) and primers R2 and SA identify mutant allele of LXRα S196A global knock-in mice (656-bp).

### Blood glucose, plasma insulin, glucagon, lipids, lipoprotein, adiponectin, and liver lipids measurements

Blood glucose levels were measured with a blood glucometer (Contour, Bayer) at the time of harvest. Blood samples were centrifuged at 10,000 rpm for 10 min and the supernatants collected as plasma. Liver lipids were extracted using the Folch lipid extraction method (chloroform: methanol: water, 2:1:1)^75^. Plasma insulin and glucagon levels were determined using the Mouse ultrasensitive insulin ELISA kit (Alpco, 80-INSMSU-E01) and the Glucagon EIA Kit (Sigma-Aldrich, RAB0202-1KT) as per the manufacture’s instructions. Plasma adiponectin concentration was quantified using the adiponectin mouse ELISA kit (Thermo Fisher Scientific, KMP0041). Total cholesterol, triglyceride, nonesterified fatty acid, and HDL-C concentrations in plasma, and total cholesterol and triglyceride in liver lipid extract were measured using colorimetric assays (Wako Diagnostics). For plasma lipoprotein analysis: for each group, equal volume of plasma per mice were pooled. Lipoproteins were separated by fast-performance liquid chromatography (Superose 6 10/300 GL column, GE Healthcare, PA, USA) on a Shimadzu HPLC system. Following separation, fractions were collected and cholesterol content was quantified by colorimetric assays.

### Hepatic VLDL-TG secretion

To measure the hepatic VLDL-TG production, mice fasted overnight were injected intraperitoneally with a solution of poloxamer 407 (Sigma-Aldrich, 16758) in PBS at 1 g/kg of mouse body weight. Blood samples were collected immediately prior to injection and at 1, 2, 3, and 4 h^76^. Triglyceride concentration were measured as described above.

### TG-intestinal metabolism

TG-intestinal metabolism was performed on mice fasted for 4 h followed by oral gavage of 10 μl olive oil/gram of mouse body weight. Blood samples were collected at 0, 1, 2, and 4 h after the gavage, and triglyceride concentration were measured as described above.

### Body fat composition

We assessed the total fat mass, lean mass, and bone mineral density (BMD) of mice after 10 weeks of western diet feeding using the LUNAR PIXIMUS bone densitometer (Lunar Corp.). Mice were anesthetized by continuous-inhalation of isoflurane and placed in a prone position on the detector tray to scan the entire mouse body. This non-invasive technique provides quantitative data on the fat tissue content, the lean tissue content, and BMD.

### Histochemical analyses

To measure lesions in the aortic root, the heart and proximal aorta were excised and the apex and lower half of the ventricles were removed. Tissues were sectioned at 6 μm intervals using a cryostat (for frozen blocks) or a microtome (for paraffin blocks). For frozen tissue sections, slides were fixed in acetone for 10 min at −20 °C. For paraffin sections, slides were dewaxed in xylene, rehydrated and boiled for 20 min in citrate buffer (10 mM, pH 6.0) for antigen retrieval. After washing three times with phosphate-buffered saline, tissue sections were incubated with primary antibodies diluted in blocking solution (10% BSA and horse serum in PBS) overnight at 4 °C in a humidified chamber. Aortic roots frozen in OCT were stained for CD68 (1:250, MCA1957, Biorad). Sections were washed and incubated with the appropriate biotinylated secondary antibodies (1:1,000, Vector laboratories), followed by washing and visualization using Vectastain ABC kit (Vector Laboratories) for CD68 or followed by horseradish peroxidase streptavidin (Vector Laboratories) and visualized using the DAB (3,3′-diaminobenzidine) peroxidase substrate kit (Vector Laboratories). The reaction mixture was counterstained with hematoxylin. BAT, liver, and pWAT were also stained for hematoxylin and eosin (H&E). Aortic root sections were stained for collagen content using the Pico Sirus Red Stain for 90 min following the manufacturer instructions (Poly Sciences), and imaged under linear polarized light with a Zeiss AxioPlan Upright. ORO staining was used to detect lipid accumulation in aortic root plaques. Formaldehyde-fixed frozen sections of the aortic arch were stained for 15 min in 0.3% ORO (Sigma-Aldrich) dissolved in 60% isopropanol and then counterstained with hematoxylin (Vector Laboratories). BAT, liver, and pWAT were stained for hematoxylin and eosin (H&E). BAT was stained for UCP1 (1:300, ab23841, Abcam), liver was stained for Clec4f (1:50, MAB2784-SP, R&D Systems), and pWAT was stained for F4/80 (1:200, MCA497RT, Biorad). Microscopic images were taken at 20×, 10×, or 4× using the EVOS FL COLOR system (Thermo Fisher Scientific). Plaque and CD68 staining morphometric measurements were performed using Image Pro Plus software (Micro Optical Solutions). Quantification of Clec4f^+^ cells in the liver was measured with Image J software. Measurement of adipocyte size in pWAT and lipid droplets in the liver was performed by automatic counting and area measurement with Image J software using the MRI adipocyte tool.

### Immunofluorescence of tissues

Recruited Ly6C^high^ EdU positive cells in the aortic root plaques were detected using Click-iT EDU Imaging Kit (MP 10338, Invitrogen). Recruitment of Ly6C^low^ beads were counted in the plaques using a fluorescent microscope, EVOS FL COLOR (Thermo Fisher Scientific). Apoptosis in the plaque was analyzed by staining the plaques with anti-cleaved caspase 3 (1:100, 9664, Cell signaling). Cell proliferation was analyzed by staining the plaques for Ki67 (1:100, ab1667, Abcam). Alpha smooth muscle actin (smooth muscle cells marker) was measured with SMaA-AF488 (1:100, 53-9760-82, Invitrogen). Aortic root plaques were also stained for DAPI (ProLong™ Gold Antifade Mountant with DAPI, P36931, Thermo Fisher Scientific). Number of positive cells was measured for each mouse.

### Hematology parameters by flow cytometry

Blood was collected before sacrificing the mice by tail bleeding. Total white blood cells (WBCs) were determined using the Genesis Hematology System (Oxford Science, Oxford, CT). Red blood cells were lysed with red blood cells lysis buffer (Sigma-Aldrich) and cells were resuspended in 2% Fc Block (553142, BD Pharmingen) for 30 min. Cells were stained with Percp/Cy5.5 anti-mouse CD45 (103132, Biolegend), PE anti-mouse CD115 (135505, Biolegend), and APC anti-mouse Ly6C/6G (108412, Biolegend). Monocytes and neutrophils were identified by flow cytometry using a LSRII analyzer and analyzed using FlowJo v10.

### Aortic digestion

Freshly-collected aortic arches were minced and digested in a LPS-depleted collagenase mixture (Liberase^TM^, Roche Applied Science) at a concentration of 0.05 mg/ml, 0.1 mg/ml of hyaluronidase (Sigma-Aldrich), 1 μM Ca^2+^ (calcium dichloride, Sigma-Aldrich), and 50 units/ml DNase I (Sigma-Aldrich), and the samples were incubated at 37 °C in a rotating shaker for 15 min. The samples were filtered through a 70-μm nylon cell strainer (Corning). The suspension was centrifuged at 1000 × *g* for 10 min, and the pelleted cells were collected, resuspended in red blood cells lysis buffer (Sigma-Aldrich) and incubated at room temperature for 5 min and washed in PBS. Cells where then processed for immune cells profiling by flow cytometry.

### Separation of stromal vascular cells (SVC)

Isolation was performed as described by Weisberg et al.^11^. Perigonadal fat pads were minced, placed in DMEM supplemented with 10 mg/ml fatty acid-poor BSA, and centrifuged at 1000 × *g* for 10 min. A LPS-depleted collagenase mixture (Liberase^TM^, Roche Applied Science) at a concentration of 0.03 mg/ml and 50 units/ml DNase I (Sigma-Aldrich) was added to the tissue, and the samples were incubated at 37 °C in a rotating shaker for 45 min. Then, the samples were passed through a 70-μm nylon cell strainer (Corning). The suspension was centrifuged at 1000  × *g* for 10 min, and the pelleted cells were collected as the SVC. The floating cells were collected as the adipocytes. The SVC were resuspended in red blood cells lysis buffer (Sigma-Aldrich) and incubated at room temperature for 5 min and washed in PBS.

### Immune cell profiling

Aortic cell suspension and SVC were resuspended in 2% Fc Block (553142, BD Pharmingen) and blocked for 30 min. Then fluorophore-conjugated primary antibodies were incubated for 30 min: PE/Cy7 anti-mouse CD45 (102114, Biolegend), APC anti-mouse F4/80 (MCA497APC, Biorad), PE anti-mouse CD11b (RM2804, Invitrogen), PE-Texas Red anti-mouse CD11c (MCD11C17, Invitrogen), BV421 anti-mouse IAIE (107631, Biolegend), BUV395 anti-mouse B220 (56793, BD Biosciences), BV786 anti-mouse CD3 (564379, BD Biosciences), PerCP/Cy5.5 anti-mouse CD4 (100540, Biolegend), APC-Cy7 anti-mouse CD8 (557654, BD Biosciences), and BV510 anti-mouse CD25 (740106, BD Biosciences) in FACS buffer (1X HBSS (Thermo Fisher Scientific), 2% BSA and 0.5 mM EDTA). All antibodies were been used at 1:200. After one wash in FACS buffer, cells were resuspended in FACS buffer containing 1 µg/mL DAPI (Invitrogen). Immune cells profiling was performed using a FACSAria II cell sorter (BD Biosciences) and data were analyzed with FlowJo v10. Adipose tissue macrophages (FB and FBC) were sorted and collected.

### Laser capture microdissection

6 µm sections of aortic roots were collected on Pen membrane Frame Slides (Arcturus). CD68^+^ cells were isolated from atherosclerotic plaques by laser capture microdissection performed under RNase-free conditions^29,77^. Aortic root sections were stained with hematoxylin-eosin and cells were captured from approximately 36 frozen sections. After laser capture microdissection, RNA was isolated using the PicoPure RNA isolation kit (Thermo Fisher scientific), and quality and quantity were determined using an Agilent 2100 Bioanalyzer (Agilent Technologies). RNA seq libraries preparation and sequencing were performed as described below.

### RNA seq samples preparation and analysis

pWAT FBC, FB RNA was extracted with RNeasy micro kit (Qiagen), plaque and pWAT T cells RNA was extracted with PicoPure RNA isolation kit (Thermo Fisher scientific), and quality and quantity were determined using an Agilent 2100 Bioanalyzer (Agilent Technologies). RNA seq libraries were prepared using the Clontech SMARTer Stranded Total RNA Seq Kit - Pico Input Mammalian following the manufacturer’s protocol. Libraries were purified using AMPure beads, pooled equimolarly, and run on a HiSeq 2500 (plaque CD68^+^ samples) or HiSeq 4000 (Plaque T cells and pWAT FBC, FB and T cells samples), paired end reads. FASTQ files were obtained, and low-quality bases as well as adapter sequences were trimmed using Cutadapt 1.18. Reads were subsequently mapped to the Mus musculus GRCm38 transcriptome using Kallisto 0.46.2. Raw counts per transcript were summed up at the gene level, and differential expression analysis was performed using edgeR 3.28.0. Genes with a *P* value < 0.05 and logFC > 0.6, or FDR < 0.05 and logFC > 1 between conditions were determined to be differentially expressed. Heatmaps were generated using heatmap 1.0.12. Pathways analysis was performed using Ingenuity Pathway Analysis (IPA, Qiagen).

### RT-qPCR of adipocytes

Total RNA was extracted from adipocytes with TRIzol (Invitrogen) and was reverse transcribed into cDNA using the Verso cDNA Synthesis Kit (Thermo Fisher scientific) according to the manufacturer’s instructions. Quantitative real-time PCR was performed on the QuantStudio 6 Flex (Applied Biosystems) using SYBR Green Fast Master Mix (Applied Biosystems). Gene-expression levels were calculated using the ΔΔCt method after their normalization to the expression levels of Cyclophilin A1 (F: 5′-GGCCGATGACGAGCCC-3′, R: 5′-TGTCTTTGGAACTTTGTCTGCAA-3′). The sequences of the mouse primers used for qPCR are: Adipoq F: 5′-TGTTCCTCTTAATCCTGCCCA-3′, 5′- CCAACCTGCACAAGTTCCCTT-3’; Fabp4 F: 5′- AAGGTGAAGAGCATCATAACCCT-3′, R: 5′-TCACGCCTTTCATAACACATTCC-3′; Plin2 F: 5′- TCTGCGGCCATGACAAGTG-3′, R: 5′-GCAGGCATAGGTATTGGCAAC-3′; Atgl F: 5′- ATGTTCCCGAGGGAGACCAA-3′, R: 5′-GAGGCTCCGTAGATGTGAGTG-3’; Hsl F: 5′- GATTTACGCACGATGACACAGT-3′, R: 5′- ACCTGCAAAGACATTAGACAGC-3’.

### Metabolic cage procedures

Energy balance phenotyping was performed using the PhenoMaster automated home cage phenotyping system (TSE Systems, Bad Homberg, Germany). The system comprises metabolic cages enclosed within a medium TSE air-cooled climate chamber (2018) that was set to maintain consistent environmental temperature (22 °C) and humidity (50%), and control the circadian light cycle (12:12; lights on at 06:30). The metabolic cage bottoms were identical to the animals’ standard housing conditions (GreenLine IVC; Techniplast, West Chester, PA). Mice were singly housed, and acclimated for 48 h within the metabolic cages. Mice had ad libitum access to food and water. A frame of infrared beams positioned along the *x* (lower long side), *y* (lower short side), and *z* (upper long side) axes of the cages enabled continuous assessment of activity levels. Repeated breakage of the same beam defined fine movement of the animal while consecutive disruption of adjacent beams defined locomotor activity. Oxygen (O2) consumption and carbon dioxide (CO2) production were measured via open-circuit indirect calorimetry (LabMaster, TSE Systems, Bad Homberg, Germany). A constant flow rate of 0.35 L/min and sample flow rate of 0.25 L/min was used, with sensors sampling air from each cage once every 30 min. Rates of O2 consumption (VO2; mL/h) and CO2 production (VCO2; mL/h) were calculated by TSE software, using the empty reference cage to provide an adjusted index of oxygen and carbon dioxide inflow. These rates were used to calculate the respiratory exchange ratio (RER = VCO2/VO2) and energy expenditure (EE = [(3.941*VO2) + (1.106*VCO2)]/1000; kcal/h). Resting energy expenditure was calculated from the average of the five lowest EE recordings for each animal during the light-phase. All measurements were obtained across two consecutive circadian cycles (48H) after the acclimatization period.

### Zymosan A-induced peritonitis

C57BL/6 WT or S196A mice were administered 100 µg zymosan A (Sigma-Aldrich) in PBS. After 4, 24, or 48 h mice were sacrificed and peritoneal exudates collected by lavage with 10 ml of ice-cold sterile PBS with 2 mM EDTA. Cellular composition of peritoneal exudate was determined by flow cytometry, as previously described^27^. Antibodies used to identify neutrophils (CD45^+^/CD115^-^/Ly6G^+^) and monocytes (CD45^+^/CD115^+^/Ly6C^high/low^) were APC anti-mouse Ly6G/Ly6C (108412, Biolegend), PE anti-mouse CD115 (135505, Biolegend), and PerCP/Cy5.5 anti-mouse CD45 (103132, Biolegend). All antibodies were used at 1:200. Monocytes and neutrophils were identified by flow cytometry using a LSRII analyzer and analyzed using FlowJo v10.

### Bone marrow-derived macrophage cultures

Bone marrow cells were isolated by flushing cells from the femurs and tibiae of mice. Cells were differentiated into bone marrow derived macrophages (BMDMs) in 4.5 g/L glucose DMEM (Lonza) with 20% fetal bovine serum (FBS), 1% penicillin/streptomycin, and murine M-CSF (10 ng/mL; PeproTech) at 37 °C and 5% CO_2_ for 7 days. The BMDMs were treated with LPS (10 ng /mL, Sigma-Aldrich) and murine IFNγ (20 ng/mL, BD Biosciences) for M1 polarization, with murine IL4 (15 ng/mL; BD Biosciences) for M2 polarization or without treatment for control M0, for 24 h.

### Mitochondrial activity: seahorse assay

Oxygen consumption was measured in a Seahorse XF24 Analyzer (Agilent). Differentiated BMDMs were seeded at 0.25 × 10^6^ cells/well in XF24-well plates (Agilent) and polarized for M0, M1, and M2 for 24 h, as described above. Wells without cells were included as a background control. Sensor plates were calibrated overnight in a CO_2_-free incubator at 37 °C using 1 mL/well of XF calibrant solution (Agilent). After polarization, culture medium was replaced with glucose-free DMEM (Agilent) medium supplemented with 2 µM L-glutamine, 1 µM pyruvate and 10 mM glucose (Agilent) and cells were incubated 1 h in a CO_2_-free incubator at 37 °C. Injection ports were loaded with 10× injection mixes to obtain a final concentration in each well of 20 mM glucose after the first injection, 1.5 µM oligomycin after the second injection, 1.5 µM FCCP after the third injection, and 0.5 µM rotenone plus 0.5 µM antimycin A after the fourth injection. After the run, supernatants were carefully aspirated and cells were fixed with methanol and stained for DAPI (Invitrogen). Cells were imaged and counted using the CellInsight CX7 LZR High Content Analysis Platform (Thermo Fisher Scientific). OCR values were normalized to cell number for each well. A representative experiment with samples in triplicates is shown.

### Determination of mitochondrial abundance: MitoTracker

BMDMs were seeded at 1 × 10^5^ cells/well in 8 wells Nunc Lab-Tek II Chamber Slide (Thermo Scientific). The next day, cells were polarized for M0, M1, and M2 as described above. After 24 h, cells were treated with 100 nM MitoTracker Red CMXRos (Invitrogen) for 30 min at 37 °C, fixed in methanol and stained for DAPI (Invitrogen). Images were taken using an EVOS m7000 Imaging System.

### Determination of mitochondrial abundance: mitochondrial DNA measure

BMDMs were seeded at 0.6 × 10^6^ cells/mL in 12-well plates and differentiated as described above. The next day, total DNA was extracted from BMDMs using a DNeasy Blood & Tissue Kit (Qiagen) and 25 ng of DNA was used for qPCR using Fast SybrGreen Master Mix (Applied Biosystems) on a QuantStudio 6 Flex (Applied Biosystems). Mitochondrial DNA (mtDNA) expression was normalized by nuclear DNA (nDNA) expression. Sequences of primers used are mtDNA F: 5′-GTACCGCAAGGGAAAGATGA-3′; mtDNA R: 5′ACCAAGCTCGTTAGGCTTTTC-3′; nDNA F: 5′-GCCAGCCTCTCCTGATTTTAGTGT-3′; nDNA R: 5′-GGGAACACAAAAGACCTCTTCTGG-3′.

### Electron Microscopy

M0, M1 and M2 LXRα WT and S196A BMDM were fixed with 2.5% glutaraldehyde and 2% paraformaldehyde in 0.1 M cacodylate buffer, and postfixed in 1% OsO_4_ in 0.1 M cacodylate buffer with 1% potassium ferrocyanide for 1 h on ice. The cells were stained en bloc with 0.25% uranyl acetate then dehydrated in graded series of ethanol on ice followed by one wash with 100% ethanol and two washes with propylene oxide (5 min each) and embedded with EMbed 812 (Electron Microscopy Sciences, Hatfield, PA). Sections were cut at 60 nm on a Leica EM UC6 ultramicrotome, and picked up on copper grids, stained with 3% uranyl acetate for 15 min and Reynolds lead citrate stain for 5 min. Grids were viewed using a Philips CM12 TEM (Philips) transmission electron microscope and photographed using a Gatan 4k × 2.7k digital camera (Gatan Inc.).

### 3T3-L1 cell culture and differentiation

3T3-L1 cells were purchased from ATCC and cultured in 4.5 g/L glucose DMEM with 10% FBS (complete media), 1% penicillin/streptomycin (complete DMEM) at 37 °C and 5% CO2. 3T3-L1 preadipocytes were induced to differentiate by incubation with 0.5 mM IBMX (Sigma-Aldrich), 1 μg/mL insulin (Gibco), 0.25 μM dexamethasone (Sigma-Aldrich) and 2 μM rosiglitazone (Cayman Chemical Company) in complete media for 2 days. The cells were then incubated with complete media containing 10 μg/mL insulin for 3 days. Then conditioned media from M0, M1, or M2 BMDMs, collected 24 h after treatments for macrophage polarization to M1 and M2 as described above, was filtered and 1 mL of the conditioned media was added after removal of the complete media to the differentiated 3T3-L1 for 24 h. Lipid accumulation was observed by staining with ORO: cells were fixed with 4% formaldehyde for 15 min, incubated with 60% isopropanol (Fisher Chemical) for 5 min, stained with 0.3% ORO (Sigma-Aldrich) in 60% isopropanol for 30 min at room temperature and counterstained with hematoxylin (Vector Laboratories). Images were taken using the EVOS FL COLOR imaging system, and five independent experiments were used to quantify the ORO staining by automatic area measurement using Image J software. A representative experiment is shown.

### Determination of secreted cytokines

Cytokine levels in cell culture supernatants samples were determined using the Proteome Profiler Mouse Cytokine Array Panel A (R&D Systems) following the manufacturer recommendations. A representative experiment with samples in duplicates is shown.

### Cell based luciferase reporter assay

HEK293 stably expressing LXRα WT or S196A^15^ were seeded in 96-well plate at a density of 1 × 10^4^ per well. Cells were transfected with 0.1 µg LXRE luciferase reporter and 0.025, 0.05 or 0.1 µg pcDNA3-PGC1α, and 0.05 µg pcDNA3 (vector only) per well. Cells were transfected, using Lipofectamine 3000 (Invitrogen), and 48 h later were treated with 5 μM T0901317 (in DMSO) or DMSO for 24 h. Luciferase activity was measured using ONE-Glo luciferase Assay System (Promega). Luciferase activity was normalized to cell viability as measured by PrestoBlue Cell Viability Reagent (Invitrogen).

### Western blotting

pWAT and BAT western blot: Tissues were minced, and lysed in RIPA buffer (50 mM Tris HCl pH 8, 150 mM NaCl, 1%NP-40, 0.5% SDS) containing protease inhibitors and Calyculin A (Cell Signaling) using a pellet disruptor. Protein lysates were quantify using the BCA assay (Thermo Fisher Scientist), and were normalized for total protein concentration. Total protein was subjected to SDS-PAGE, transferred to PVDF membranes (Millipore), incubated with blocking solution (5% BSA in TBS pH 7.4) for 1 h and incubated overnight at 4 °C in primary antibody: Adipoq (1:1000, 2789 T, Cell Signaling), Fabp4 (1:1000, 2120 S, Cell Signaling), Atgl (1:1000, 2439 S, Cell Signaling), P-Hsl (Ser563) (1:1000, 4139 T, Cell Signaling), Hsl (1:1000, 18381T, Cell Signaling), Hsp90 (1:5000, 610419, BD Biosciences), and UCP1 (1:20000, 14670S, Cell Signaling). After secondary incubation at room temperature for 1 h, protein bands were visualized using Clarity Western ECL substrate (BioRad) using the iBright CL1500 Imaging system (Invitrogen). Quantification of western blots was done by using ImageJ software. A representative experiment is shown, *n* = 5. Full western blot membranes are showed in Supplementary Fig. 16A-B.

### Statistics and reproducibility

Statistical analyses were performed using GraphPad Prism 8.0.2 for PC. Data are reported as mean ± SD. Number of replicates (*n*) and statistical tests used are described in the figure legends, with a *p* value < 0.05 being considered significant and levels of significance denoted as *p < 0.05; **p < 0.01; and ***p < 0.001. In general, a two-tailed unpaired Student’s *t* test was used when comparing two groups, and a two-way ANOVA was used when comparing interactions between genotype (LXRα WT or LXRα S196A) and stimulus (M0, M1, M2). For in vivo experiments, *n* = number of animals. Samples were randomly allocated into experimental groups, and no data were excluded in the analysis. Experiments were repeated three times and had high reproducibility. The cytokine array from the conditioned media was repeated twice.

### Reporting summary

Further information on experimental design is available in the Nature Research Reporting Summary linked to this paper.

## Supplementary information

Supplementary Information

Description of Additional Supplementary Files

Supplementary Data 1

Supplementary Data 2

Supplementary Data 3

Supplementary Data 4

Supplementary Data 5

Reporting Summary

## Data Availability

The RNA seq data generated and analyzed in this study have been deposited in the GEO database under accession number GSE162660. The remaining data are provided within this article and its Supplementary Information files. Additional information is available from the corresponding author upon reasonable request.
