# Supplementary material for: Inhibiting LXRα phosphorylation in hematopoietic cells reduces inflammation and attenuates atherosclerosis and obesity in mice
Source: Commun Biol. 2021 Mar 26;4:420. doi: 10.1038/s42003-021-01925-5 (PMC7997930; doi:10.1038/s42003-021-01925-5)
Supplement: Supplementary file 1 — Supplementary Information [file 42003_2021_1925_MOESM1_ESM.pdf]

Supplementary Figure 1: LXRα S196A increases Clec4f staining in livers without affecting lipid accumulation, or genes expression

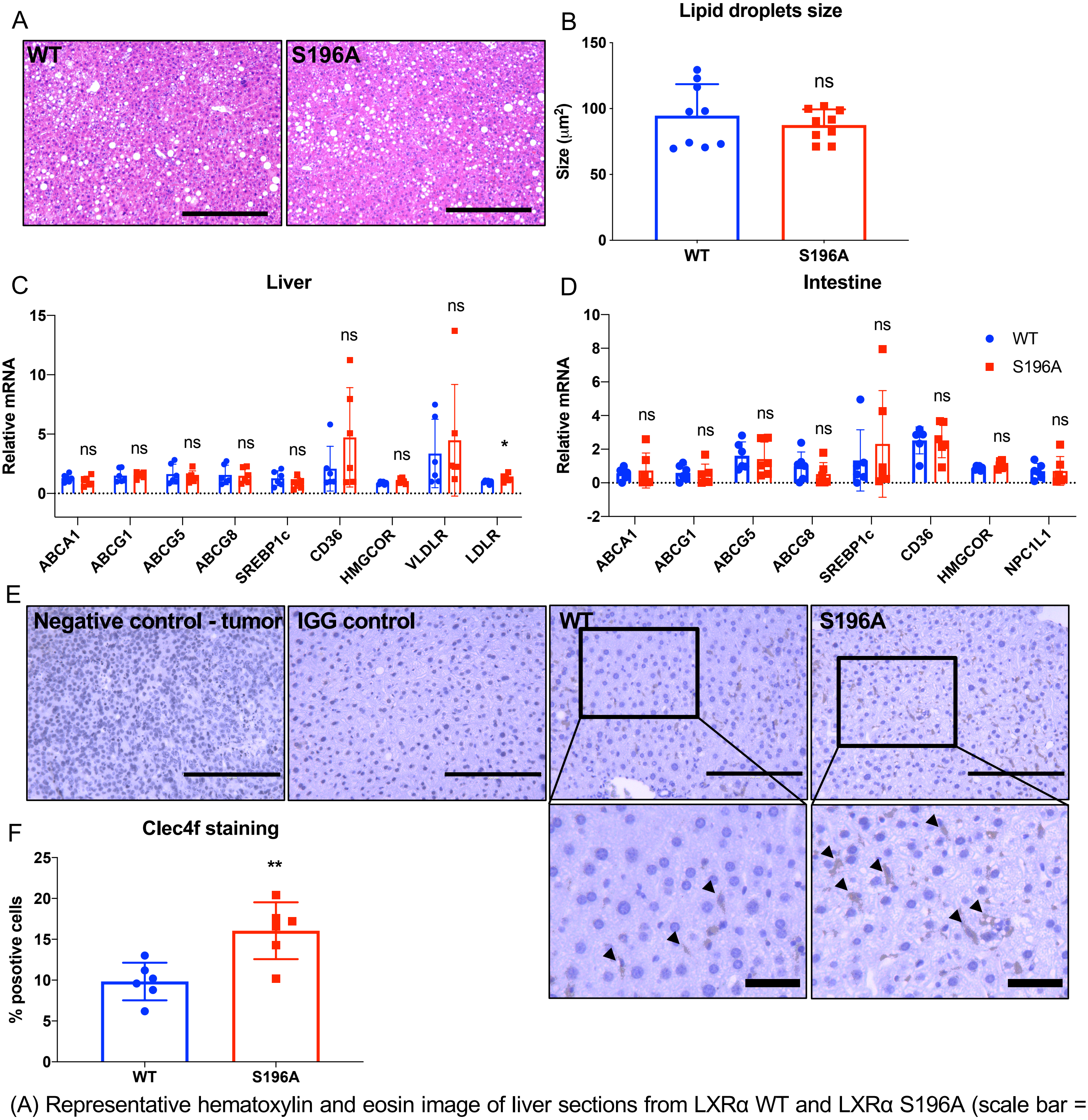

(A) Representative hematoxylin and eosin image of liver sections from LXRα WT and LXRα S196A (scale bar = 400 μm), and (B) quantification of lipid droplets in liver sections. (C-D) Expression of cholesterol homeostasis genes by qPCR: cholesterol secretion (*Abca1, Abcg1*), cholesterol excretion (*Abcg5*, *Abcg8*), fatty acid metabolism (*Srebp1c*), fatty acid uptake (*Cd36*), cholesterol biosynthesis (*Hmgcor*), and lipoproteins uptake (*Vldlr*, *Ldlr*), and intestinal cholesterol uptake (*Npc1l1*) in (C) the liver, and (D) the intestine. (E-F) Representative image of Clec4f staining, and quantification of positive cells in liver sections (scale bars: 200μm and 50μm). Data are expressed as mean ± SD (n=9 for lipid droplet quantification in liver, n=5 for gene expression, and n=7 for Clec4f quantification) and obtained from independent samples. T test; *P<0.05 and **P<0.01.

Supplementary Figure 2: LXRα S196A shows decreased TG in the liver, and full body LXRα S196A increased VLDL secretion and plasma TG clearance

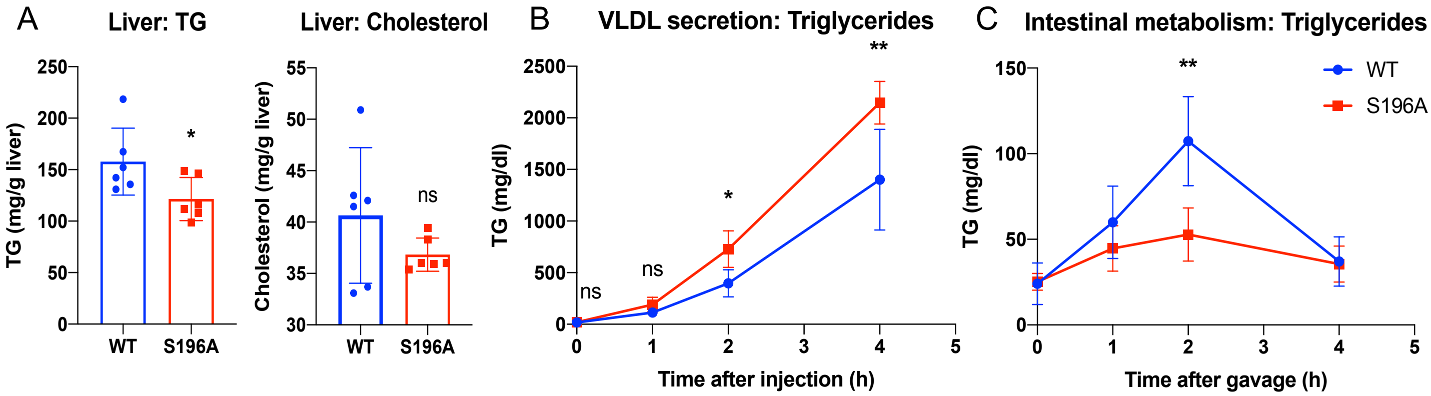

(A) Measurements of TG and total cholesterol from the livers of the *Ldlr*$^{-/-}$ LXRα WT and S196A mice on western diet for 16 weeks. (B-C) VLDL secretion and intestinal metabolism in LXRα WT and LXRα S196A mice on chow diet. (B) VLDL secretion was measured in fasted mice (4 h) by monitoring plasma TG following oral gavage of olive oil at the indicated time points. (C) Intestinal metabolism measured by TG plasma on overnight fasted mice and treated with the LPL inhibitor poloxamer 407 to inhibits lipolysis of TG-rich lipoprotein particles. Data are expressed as mean ± SD (n=6) and obtained from independent samples. T test; *P<0.05 and **P<0.01.

Supplementary Figure 3: Effect of LXRα S196A on aortic plaque characteristics

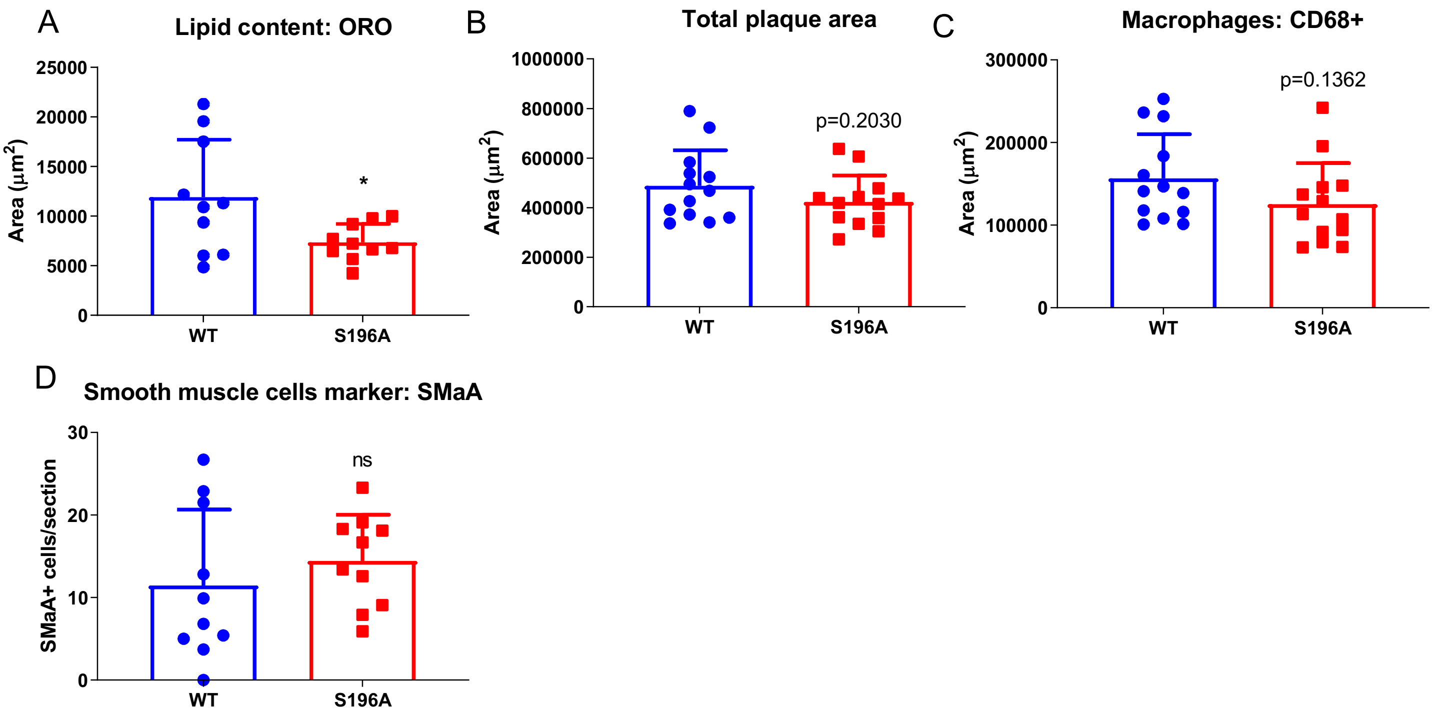

(A) Lipid content (Oil Red O), (B) total plaque area quantification, (C) area of macrophages ($CD68^+$), and (D) number of cells per section expressing SMaA (smooth muscle cell actin marker) were measured after staining of aortic sections. Data are expressed as mean ± SD (n=13 per group, except for ORO, and SMaA n=10) and obtained from independent samples. T test; *P<0.05.

Supplementary Figure 4: Effect of LXRα S196A on monocytes and neutrophils in the blood, and in the peritoneal cavity under acute inflammation

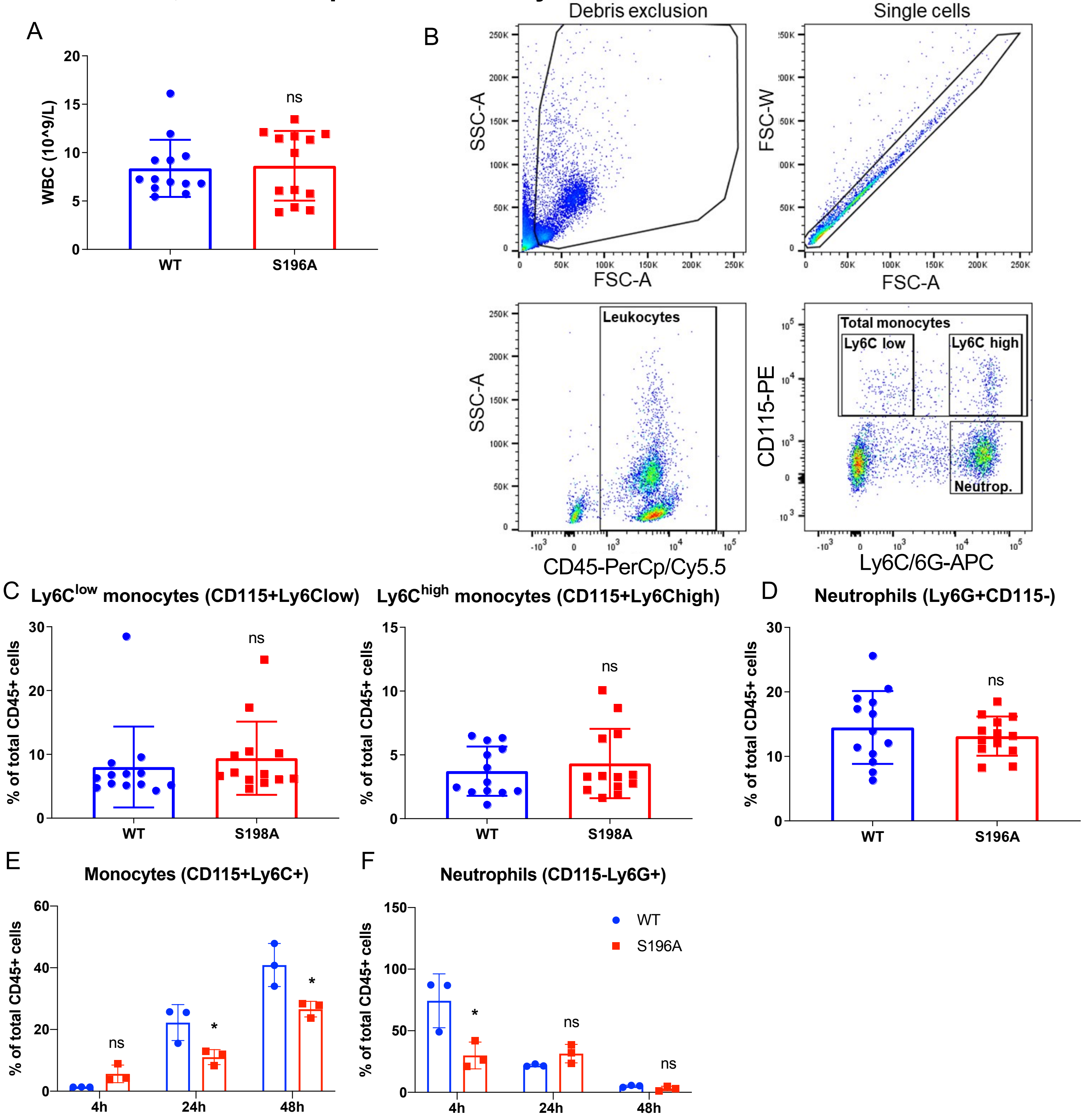

(A) Total number of white blood cells in the circulation (WBC) (WT;8.375±2.954, S196A: 8.642±3.600 10^9 cells/L, p=0.8380). (B) Gating strategy for monocytes and neutrophils quantification by flow cytometry. (C) Ly6C^{low} (WT; 8.0±6.3, S196A: 9.4±5.7 %, p=0.5640) and Ly6C^{high} monocytes (WT:3.7±1.9, S196A: 4.3±2.7 % of total CD45^{+} cells, p=0.5237), and (D) neutrophils (WT:6.3±5.7, S196A: 8.3±3.0 % of total CD45^{+} cells, p=0.4564) were measured by flow cytometry. (E-F) Full body LXRα S196A and WT mice on chow diet were injected intraperitoneally with zymosan A, and (E) monocytes and (F) neutrophils recruitment was measured at 4, 25, and 48 hours. Data are expressed as mean ± SD (n=13 per group, or n=3 for zymosan A-injected monocytes and neutrophils recruitment) and obtained from independent samples. T test; *P<0.05.

Supplementary Figure 5: Flow cytometry of immune cells in atherosclerotic plaque

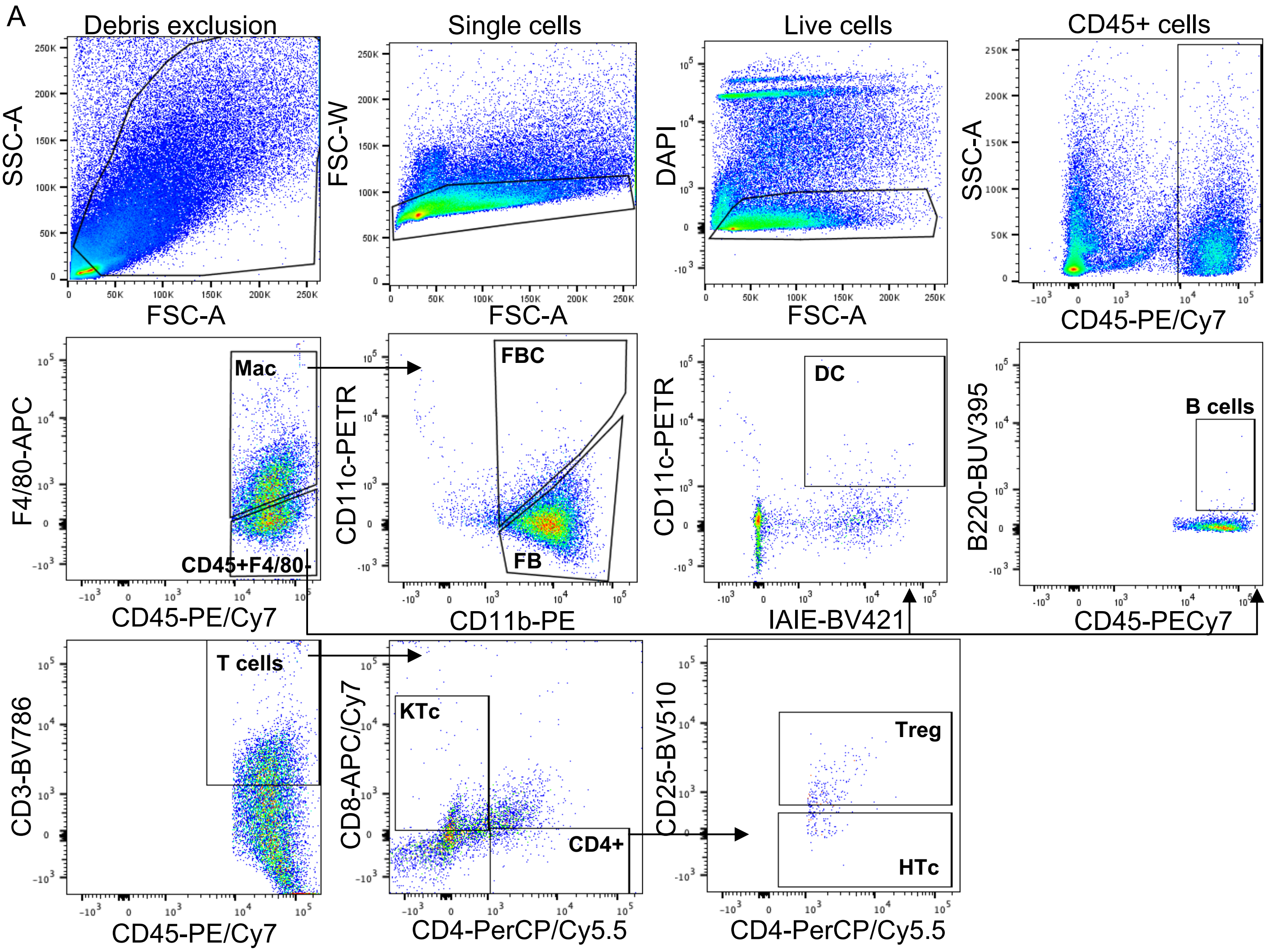

(A) Gating strategy for immune cells population from aortic digestions of atherosclerotic plaque from *Ldlr*-/- mice reconstituted with WT and S196A bone marrow and fed a western diet for 16 weeks.

Supplementary Figure 6: Effects of LXRα S196A bone marrow transplantation on immune cells in atherosclerotic plaque

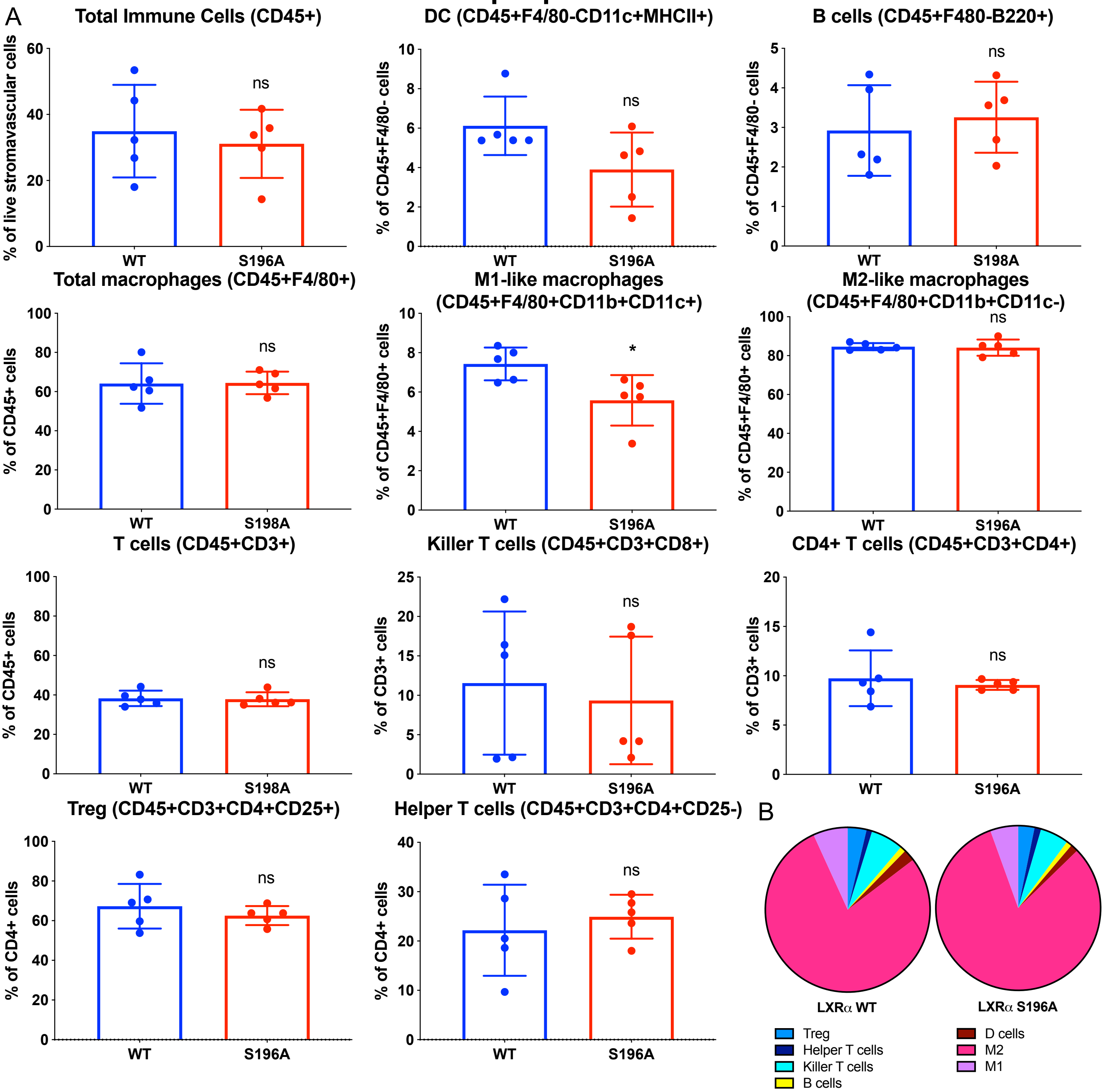

(A) Quantification of immune cells by flow cytometry in digested aortas from WT and S196A mice. Total immune cells ($CD45^+$ WT:34.94±14.02, S196A:31.12± 10.32%, p=0.6370), dendritic cells (DC WT:6.12±1.48, S196A:3.90±1.88 %, p=0.0723), B cells (Bc WT:2.92±1.15, S196A:3.26±0.90 %, p=0.6198), total macrophages ($F4/80^+$ WT:64.12±10.35, S196A:64.46± 5.76 %, p=0.9504), M1-like macrophages ($CD11b^+CD11c^+$ WT:7.43±0.83, S196A:5.58±1.28 %, p=0.0269), M2-like macrophages ($CD11b^+CD11c^-$ WT:84.60±1.83, S196A:84.10±4.17 %, p=0.8122), total T cells ($CD3^+$ WT:38.28±3.90, S196A:37.86±3.56 %, p=0.8631), Killer T cells ($CD8^+$ WT:11.55±9.09, S196A:9.35±8.08 %, p=0.6965), $CD4^+$ T cells ($CD4^+$ WT:9.75±2.82, S196A:9.07±0.50 %, p=0.6144), Treg ($CD4^+C25^+$ WT:67.30±11.27, S196A:62.58±4.77 %, p=0.4135), and Helper T cells ($CD4^+CD25^-$ WT:22.17±9.24, S196A:24.92±4.45 %, p=0.5659) populations. (B) Pie chart representing the percentage of different cell population of immune cells in the plaque. Data are expressed as mean ± SD (n=5 per group) and obtained from independent samples. T test; *P<0.05.

Supplementary Figure 7: Heatmap of plaque CD68$^+$ and T cells.

A

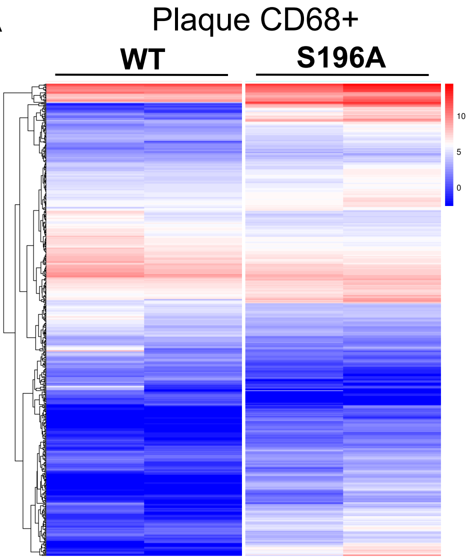

C

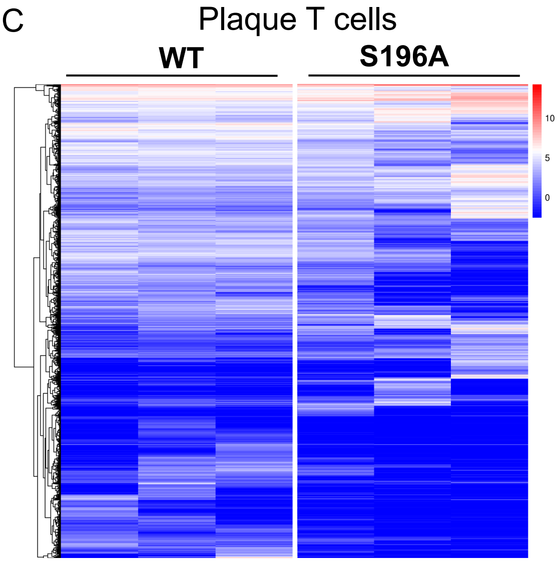

B

**Gene**	**Log(FC)**	**Gene**	**Log(FC)**
**Cox7a1**	8.54	**Atp5j2**	1.09
**Cox8b**	6.8	**Atp5b**	1.08
**Cox6a2**	6.78	**Cox7a2**	1.07
Cpt1b	4.8	**Atp5c1**	1.07
Mapk10	4.09	**Ndufb4**	1.04
**Ndufa5**	1.84	**Ndufs3**	1.04
**Atp5g1**	1.5	**Atp5a1**	0.95
**Atp5o**	1.42	Pink1	0.92
**Ndufb9**	1.41	Prdx3	0.91
**Ndufa4**	1.41	**Atp5h**	0.9
Aco2	1.38	**Ndufs1**	0.89
**Cox7b**	1.29	**Cox4i1**	0.88
Pdha1	1.29	**Ndufa13**	0.87
**Uqcrfs1**	1.25	**Atp5g3**	0.86
**Cycs**	1.2	Vdac1	0.85
**Ndufb11**	1.18	**Ndufs2**	0.83
**Cox6c**	1.17	**Atp5l**	0.62
**Atp5j**	1.11	Ogdh	0.62

(A) Heatmap of plaque CD68$^+$ cells captured by LCM from LXRα WT and S196A by RNA seq: 570 significant genes; LogFC> 0.6; pvalue<0.05). (B) Genes and fold change of upregulated mitochondrial function-associated genes in CD68$^+$ RNA seq. Genes in bold are involved in mitochondrial oxidative phosphorylation pathway. (C) Heatmap of plaque T cells collected by flow cytometry from LXRα WT and S196A by RNA seq: 3970 significant genes; LogFC> 1; FDR<0.05).

Supplementary Figure 8: Effects of LXRα S196A bone marrow transplantation on diet induced obesity

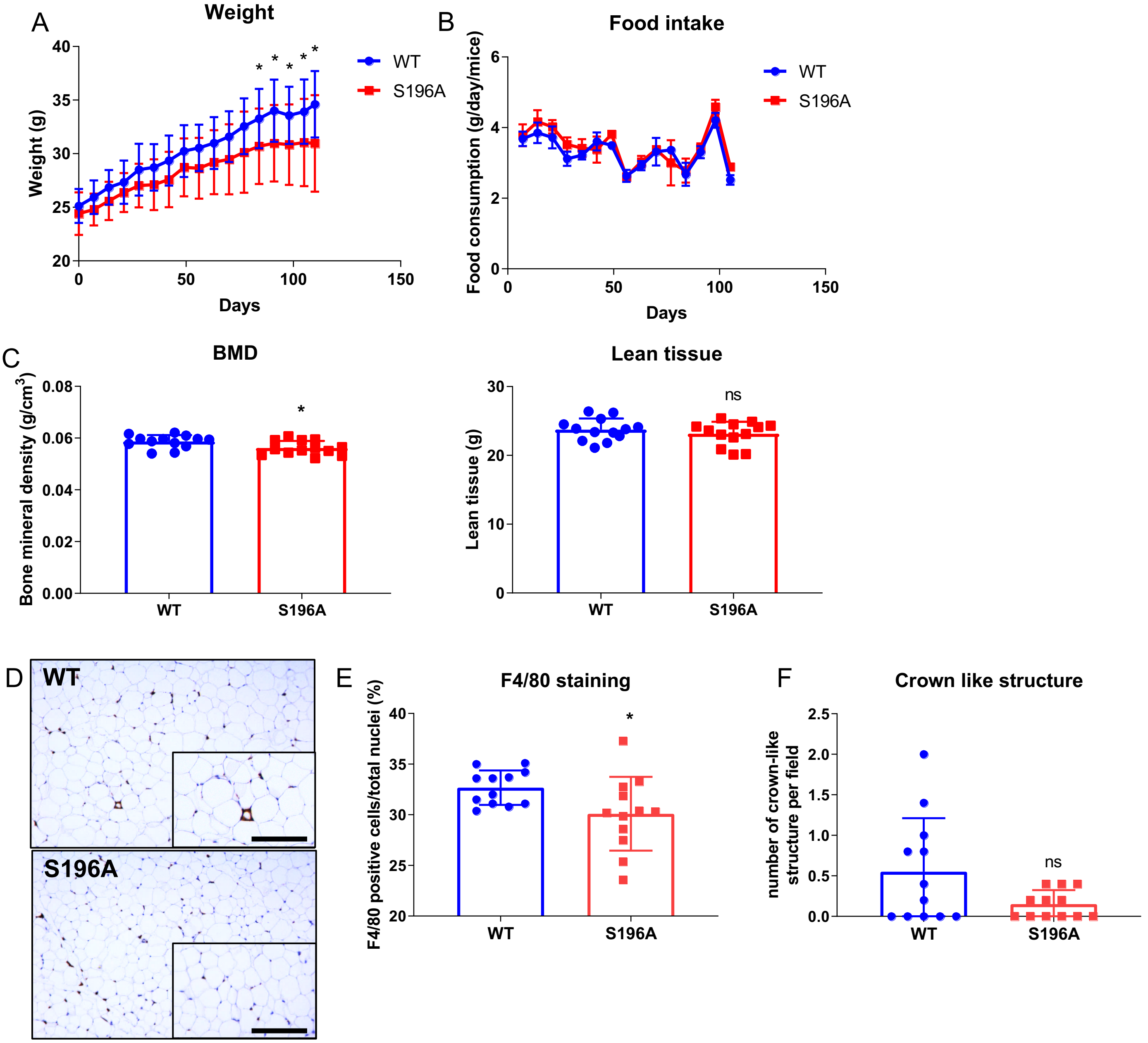

(A) Mouse body weight, (B) food intake, (C) bone mineral density and lean tissue quantification by DEXA scan were determined from *Ldlr-/-* mice reconstituted with bone marrow from WT and S196A mice. (D) Representative F4/80 staining images (scale bar = 400 µm), (E) quantification of F4/80 staining and (F) of crown-like structures of pWAT sections. Data are expressed as mean ± SD (n=13 per group except for F4/80 staining and crown-like structure n=12) and obtained from independent samples. T test; *P<0.05.

Supplementary Figure 9: LXRα S196A increases adiponectin levels but does not affect UCP1 expression in BAT

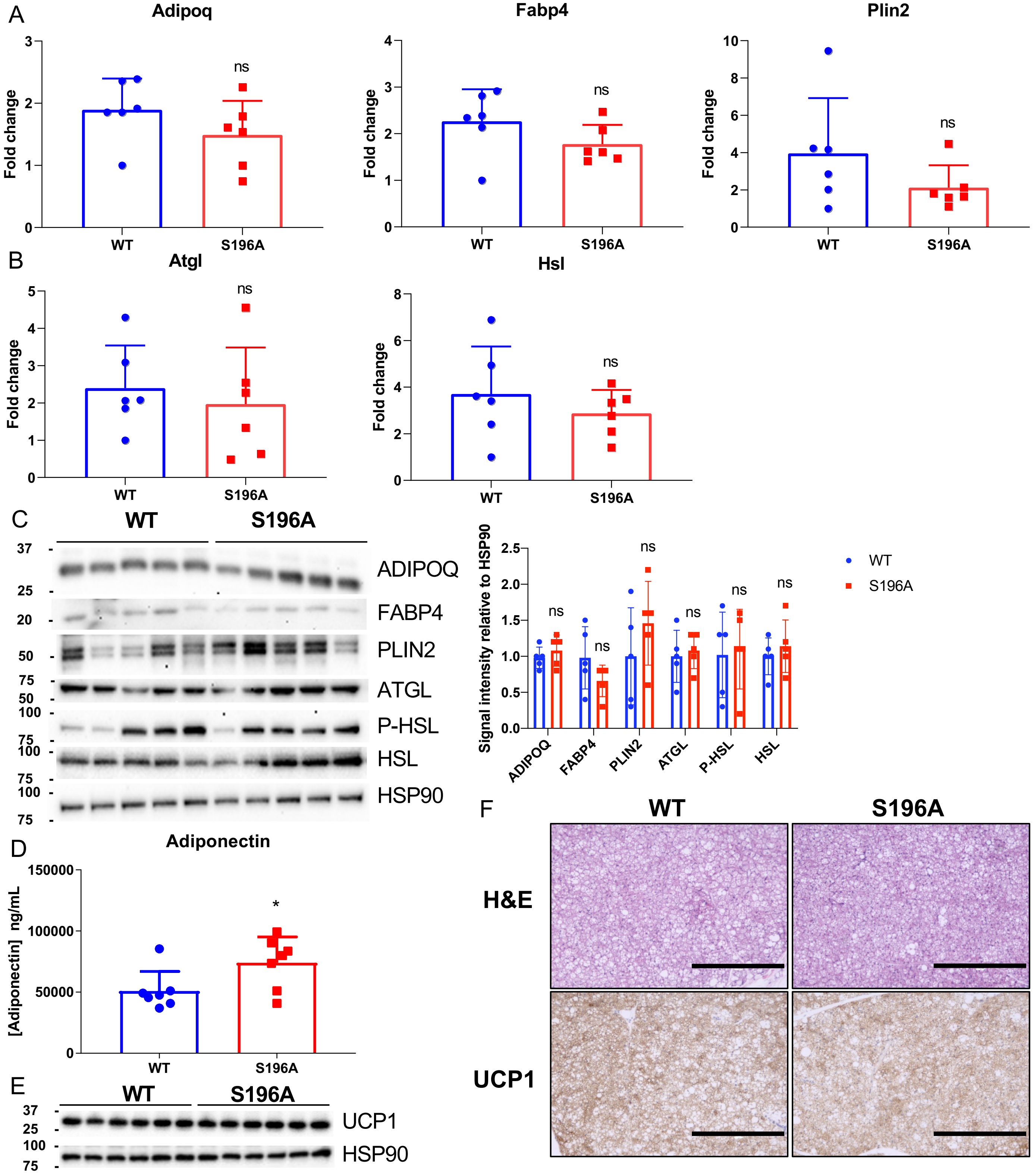

(A) Gene expression of adipocyte differentiation marker in pWAT adipocytes: *Adipoq* (Adiponectin), *Fabp4* (Fatty acid binding protein 4) and P*lin2* (Perilipin2), and (B) lipolysis activators: *Atgl* (Adipose triglyceride lipase) and *Hsl* (Hormone-sensitive lipase). (C) pWAT protein expression assessed by western blot and quantification of the adipocyte differentiation markers. (D) Plasma adiponectin level. Protein expression of UCP1 in brown adipose tissue (BAT) by (E) western blot, and (F) immunohistochemistry. Representative image of hematoxylin and eosin and UCP1 staining in BAT (scale bars: 400μm). Data are expressed as mean ± SD (n=6 for gene expression and BAT protein expression, n=5 for pWAT protein expression, and n=7 for adiponectin level) and obtained from independent samples. T test; *P<0.05.

Supplementary Figure 10: LXRα S196A does not affect activity or energy expenditure but increases the respiratory exchange ratio in the dark cycle

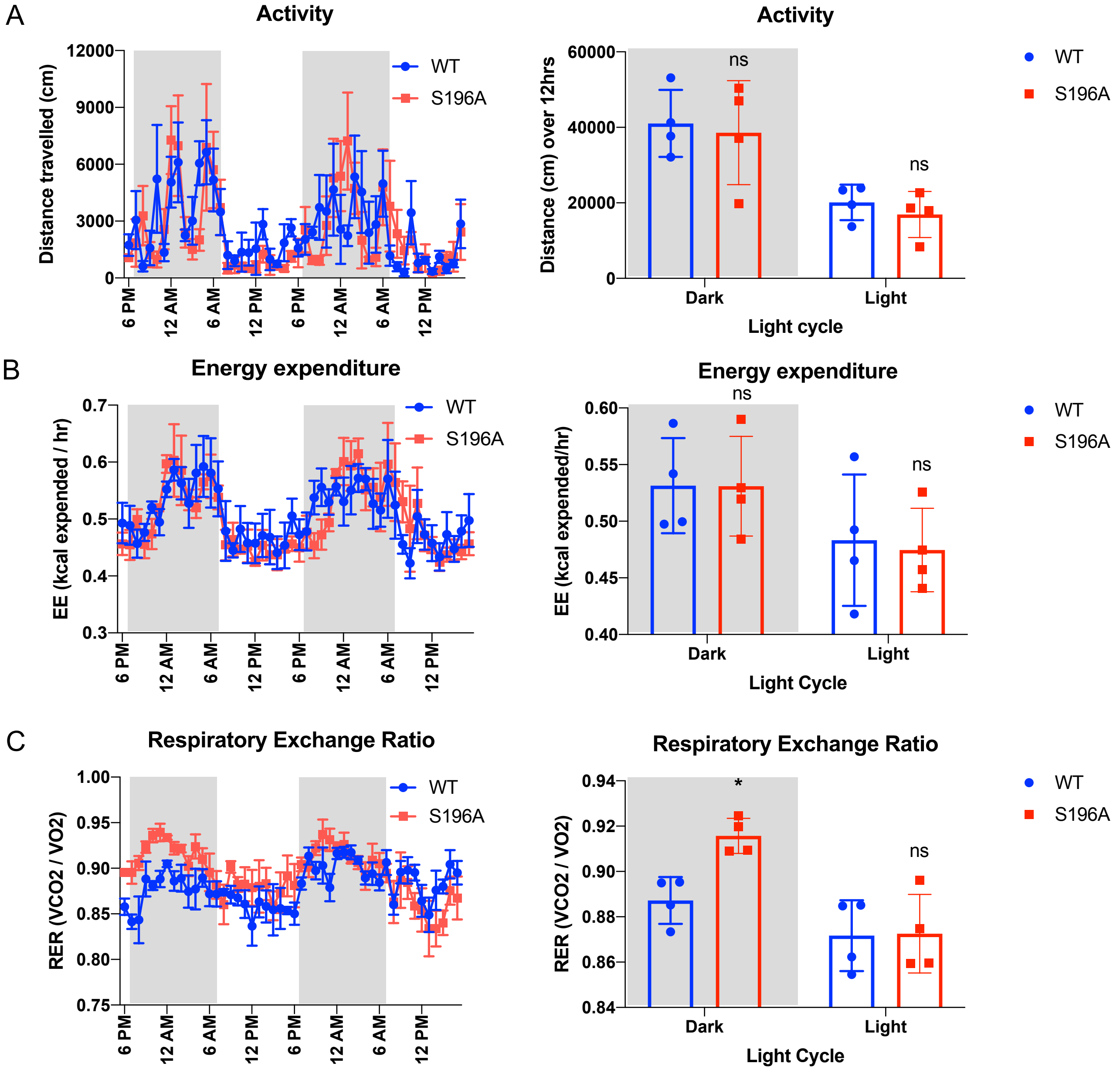

(A) Activity, (B) energy expenditure (EE), and (C) respiratory exchange ratio (RER) in LXRα WT or S196A fed a western diet for 16 weeks. Data are expressed as mean ± SD (n=4) and obtained from independent samples. T test; *$P<0.05$.

Supplementary Figure 11: Flow cytometry of immune cells in pWAT

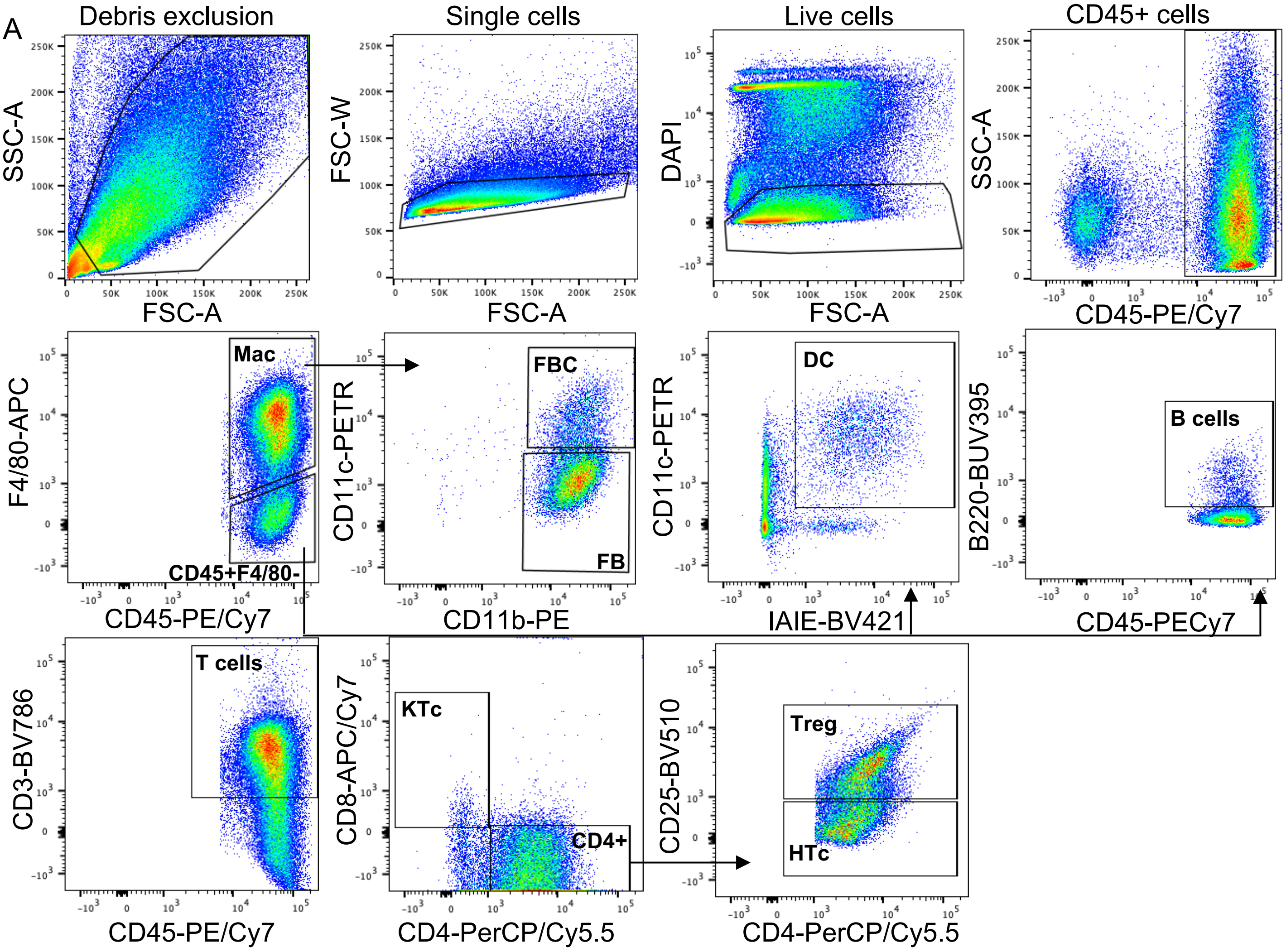

(A) Gating strategy for immune cells population in pWAT from mice expressing WT or S196A from the bone marrow.

Supplementary Figure 12: Effects of LXRα S196A on immune cells in adipose tissue

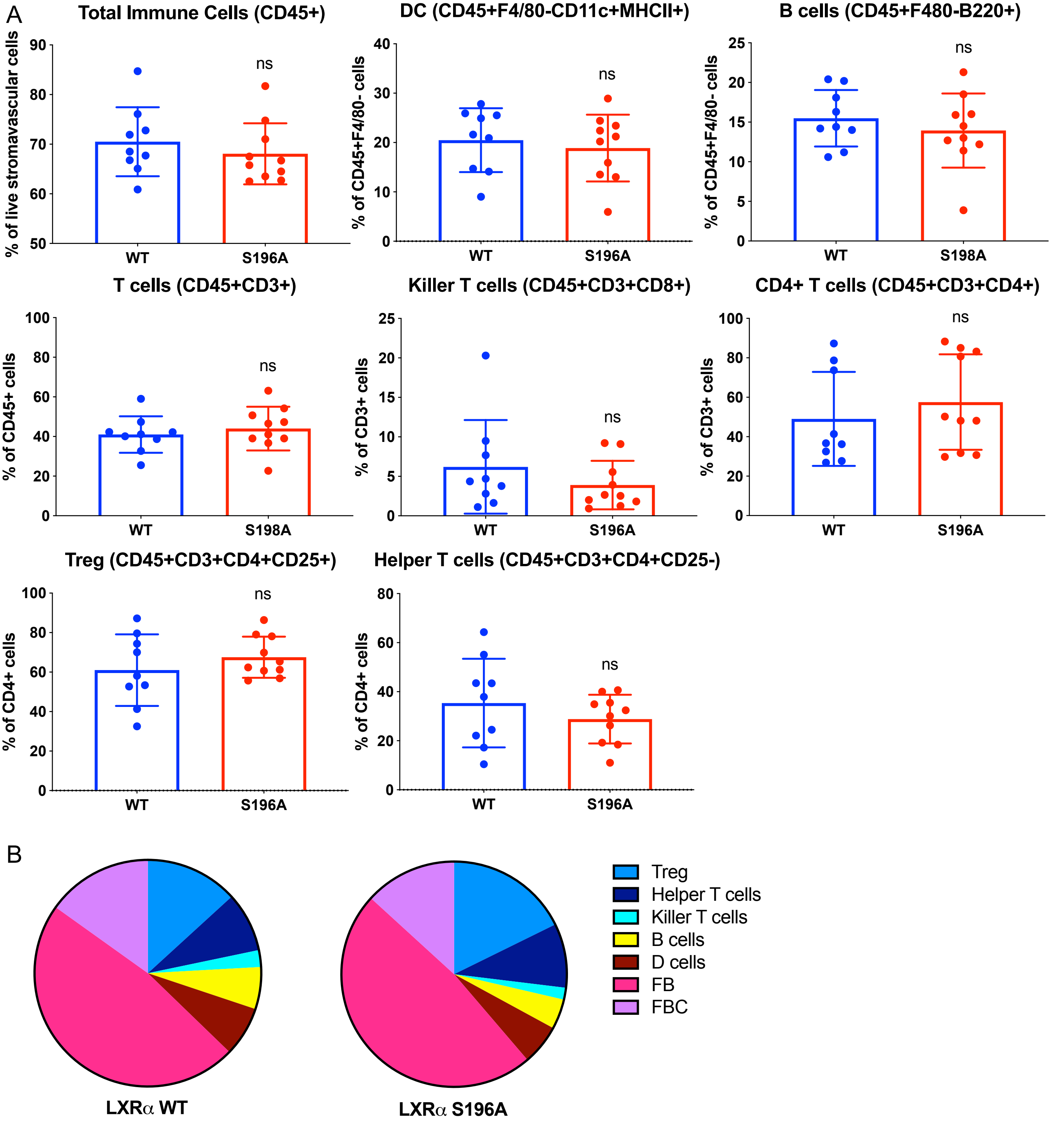

(A) Quantification of immune cells by flow cytometry in digested pWAT from *Ldlr*$^{-/-}$ mice reconstituted bone marrow from WT and S196A mice. Total immune cells ($CD45^+$ WT:70.50±6.96, S196A:68.07±6.15 %, p=0.4300), dendritic cells (DC WT:20.49±6.46, S196A:18.88±6.75%, p=0.6038), B cells (Bc WT:15.49±3.55, S196A:13.94±4.672%, p=0.4310), total T cells ($CD3^+$ WT:41.00±9.24, S196A:44.02±11.04%, p=0.5292), Killer T cells ($CD8^+$ WT:6.21±5.93, S196A:3.90±3.07%, p=0.2947), $CD4^+$ T cells ($CD4^+$ WT:49.01±23.84, S196A:57.58±24.23%, p=0.4487), Treg ($CD4^+C25^+$ WT:61.02±18.13, S196A:67.57±10.41%, p=0.3414), and Helper T cells ($CD4^+CD25^-$ WT:35.38±18.07, S196A:28.83±9.92 %, p=0.3343) populations. (B) Pie chart representing the percentage of different populations of immune cells in the pWAT. Data are expressed as mean ± SD (n=9/10 per group) and obtained from independent samples. T test.

Supplementary Figure 13: LXRα S196A ATMs regulate genes in pWAT T cells

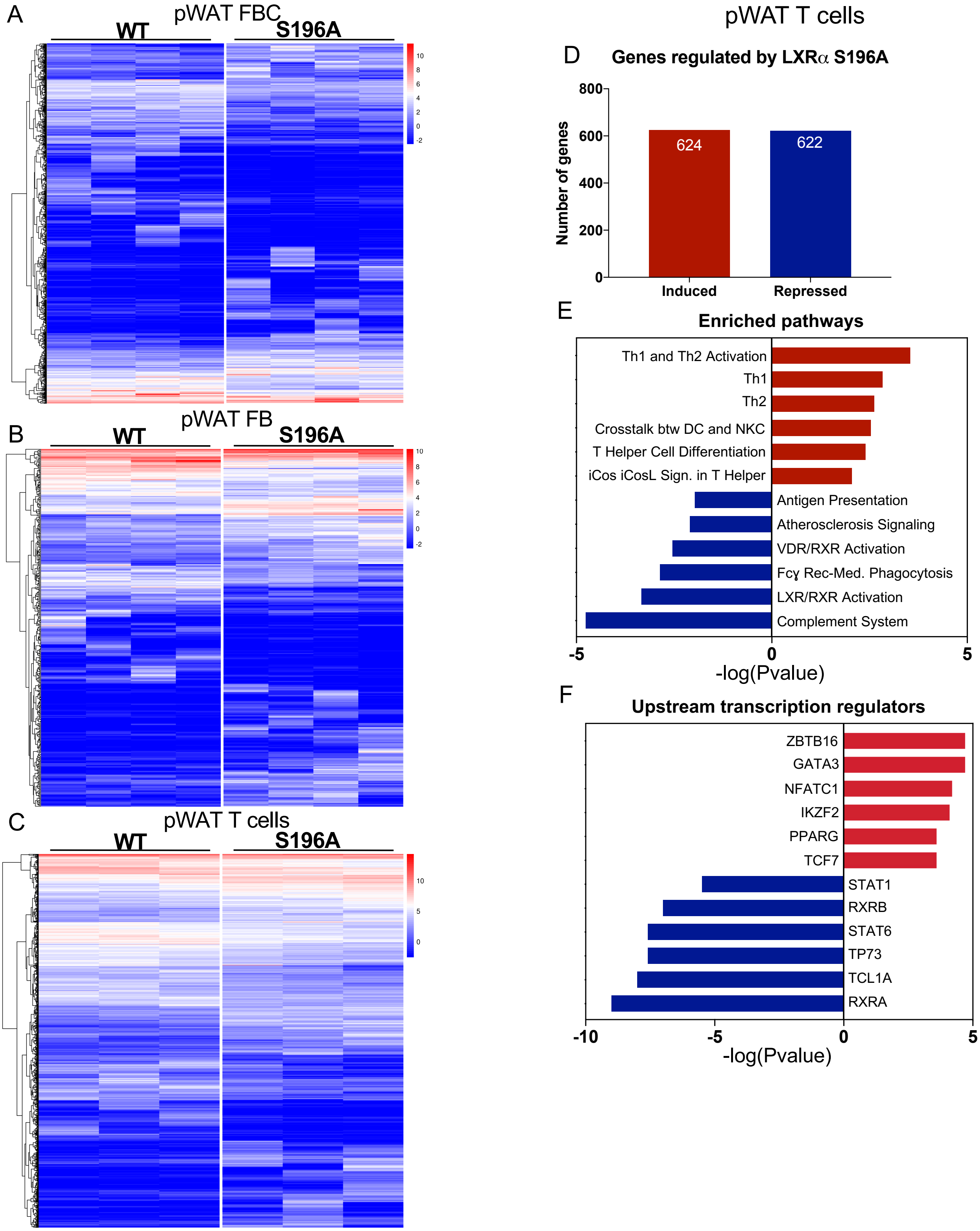

RNA seq analysis of pWAT T cells sorted by flow cytometry from LXRα S196A vs WT mice. (A) Heatmap of pWAT FBC cells from LXRα WT and S196A (RNA seq: 798 significant genes; LogFC > 1, FDR < 0.05). (B) Heatmap of pWAT FB cells from LXRα WT and S196A (RNA seq: 410 significant genes; LogFC > 1, FDR < 0.05). (C) Heatmap of pWAT T cells from LXRα WT and S196A (RNA seq: 1246 significant genes; LogFC > 1, FDR < 0.05). (D) Number of genes regulated in pWAT T cells by LXRα S196A versus WT (LogFC > 1, FDR < 0.05). (E) Enriched pathways in pWAT Y cells in S196A after DGE analysis with IPA. (F) Transcriptional regulators controlling the upregulated or downregulated genes in pWAT T cells.

Supplementary Figure 14: Steady state blood glucose, insulin and glucagon levels in LXRα WT and S196A mice.

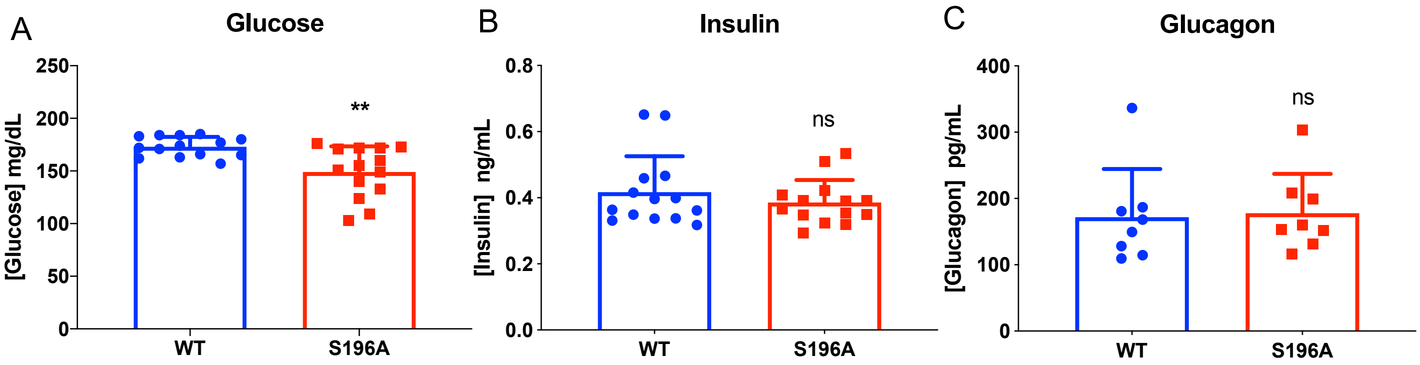

(A) Steady-state blood glucose levels from WT and S196A mice on western diet for 16 weeks was determined. Steady state (B) insulin and (C) glucagon levels were also determined from the same cohort of mice as the blood glucose measurements. Data are expressed as mean ± SD (n=14 for glucose and insulin, and n=8 for glucagon) and obtained from independent samples. T test; **P<0.01.

Supplementary Figure 15: Model for T cell-macrophage cross talk in the plaque of S196A mice

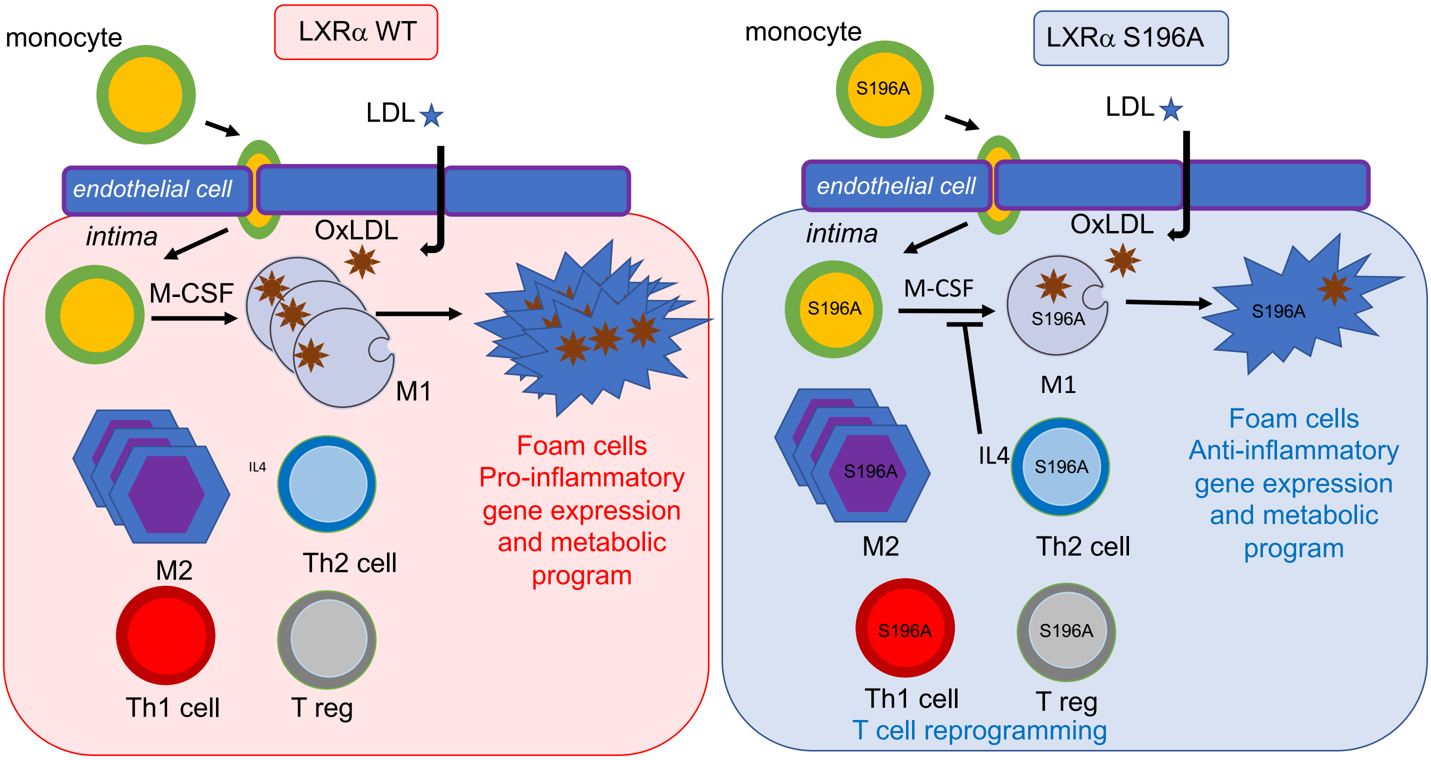

Mice expressing WT LXRα results in macrophage proliferation and atherosclerosis. In bone marrow transplant of S196A, where both innate and adaptive immune cells express S196A, we suggest that signals from T cells, such as IL4 from Th2 cells, are increased in S196A compared to WT, which can antagonize macrophage proliferation, promote a less inflammatory gene expression and metabolic phenotype in the plaque, thereby attenuating atherosclerosis. In the monocyte specific S196A mouse model in Gage *et al.*[17], the increased expression of *Il4* from T cells would not occur because it expresses WT LXRα rather than LXRα S196A, and macrophage proliferation would not be restrained, therefore intensifying atherosclerosis. We have not included dendritic cells or B cells for simplicity. We posit similar effects could also be occurring in T cells in VAT that express LXRα S196A to restrain inflammatory macrophage accumulation.

Supplementary Figure 16: Complete western blots from Supplementary Figure 9

A Supplementary Figure 9C - western blot pWAT

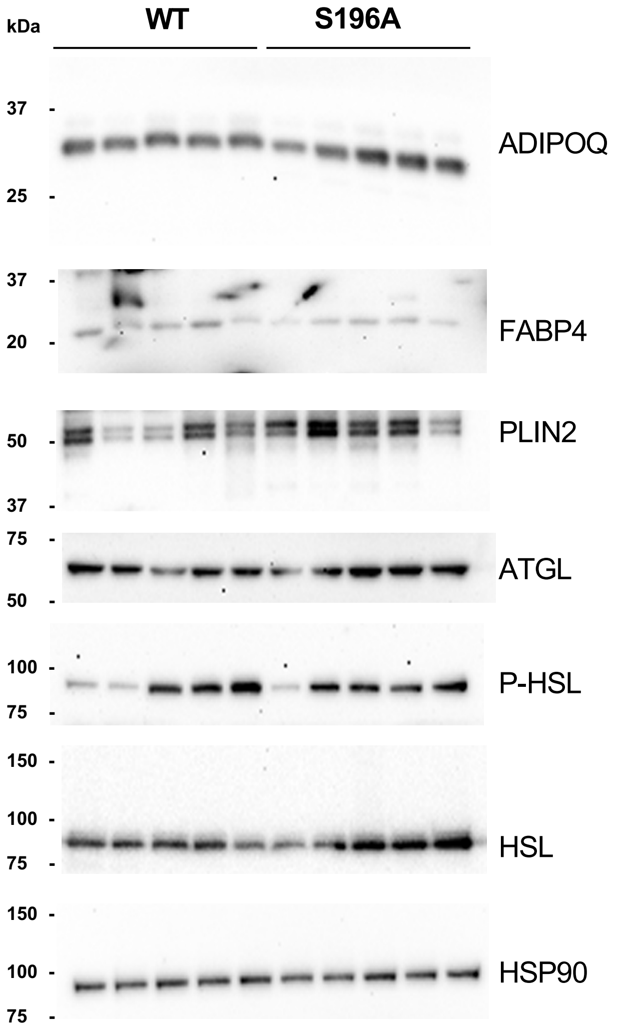

B Supplementary Figure 9E - western blot BAT

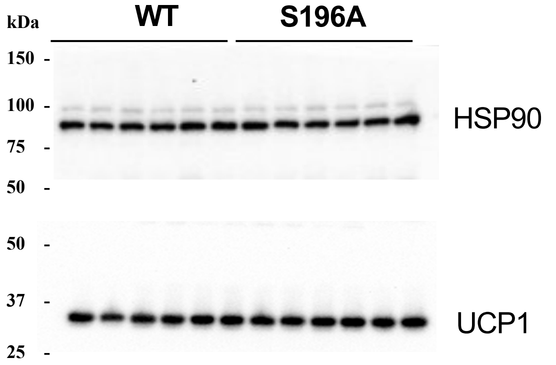

Supplementary Table 1: Genes within IPA pathways in LXRα S196A versus WT from plaque CD68⁺ cells

Mito. Function	
Gene	LogFC
Cox7a1	8.54
Cox8b	6.80
Cox6a2	6.78
Cpt1b	4.80
Mapk10	4.09
Ndufa5	1.84
Atp5g1	1.50
Atp5o	1.42
Ndufb9	1.41
Ndufa4	1.41
Aco2	1.38
Cox7b	1.29
Pdha1	1.29
Uqcrfs1	1.25
Cycs	1.20
Ndufb11	1.18
Cox6c	1.17
Atp5j	1.11
Atp5j2	1.09
Atp5b	1.08
Cox7a2	1.07
Atp5c1	1.07
Ndufb4	1.04
Ndufs3	1.04
Atp5a1	0.95
Pink1	0.92
Prdx3	0.91
Atp5h	0.90
Ndufs1	0.89
Cox4i1	0.88
Ndufa13	0.87
Atp5g3	0.86
Vdac1	0.85
Ndufs2	0.83
Atp5l	0.62
Ogdh	0.62

Oxphos	
Gene	LogFC
Cox7a1	8.54
Cox8b	6.80
Cox6a2	6.78
Ndufa5	1.84
Atp5g1	1.50
Atp5o	1.42
Ndufb9	1.41
Ndufa4	1.41
Cox7b	1.29
Uqcrfs1	1.25
Cycs	1.20
Ndufb11	1.18
Cox6c	1.17
Atp5j	1.11
Atp5j2	1.09
Atp5b	1.08
Cox7a2	1.07
Atp5c1	1.07
Ndufb4	1.04
Ndufs3	1.04
Atp5a1	0.95
Atp5h	0.90
Ndufs1	0.89
Cox4i1	0.88
Ndufa13	0.87
Atp5g3	0.86
Ndufs2	0.83
Atp5l	0.62

Sirtuin Signaling	
Gene	LogFC
Pgam2	6.25
Ppargc1a	5.21
Ppara	4.82
Cpt1b	4.80
Rarb	4.37
Slc25a4	2.45
Ndufa5	1.84
Idh2	1.65
Pdk1	1.56
Atp5g1	1.50
Ndufb9	1.41
Ndufa4	1.41
Pdha1	1.29
Uqcrfs1	1.25
Ndufb11	1.18
Atp5j	1.11
Atp5b	1.08
Atp5c1	1.07
Ndufb4	1.04
Ndufs3	1.04
Acadl	1.00
Got2	0.97
Atp5a1	0.95
Ndufs1	0.89
Ndufa13	0.87
Vdac1	0.85
Ndufs2	0.83

TCA Cycle	
Gene	LogFC
Mdh1	1.69
Aco2	1.38
Idh3a	1.30
Fh1	1.16
Mdh2	1.02
Cs	0.74
Ogdh	0.62

β-adrenergic Signaling	
Gene	LogFC
Ppp1r3a	6.19
Ryr2	5.70
Cacna1s	5.47
Ppp1r3c	5.28
Adcy1	4.56
Ppp1r14c	4.18
Akap6	3.47
Pde1c	2.70
Atp2a2	2.39
Ppp2r3a	1.70
Prkag2	1.06
Slc8a1	0.75

PKA Signaling	
Gene	LogFC
Myl7	13.56
Myl1	11.60
Myl4	10.06
Mylk3	8.89
Ttn	7.53
Tnni3	7.45
Ppp1r3a	6.19
Ryr2	5.70
Pygm	5.34
Ppp1r3c	5.28
Myl3	4.75
Adcy1	4.56
Ryr3	4.47
Ppp1r14c	4.18
Akap6	3.47
Pde1c	2.70
Pygb	1.09
Prkag2	1.06

NFAT in Immune cells	
Gene	LogFC
Cd79b	-3.26
Cd86	-1.68
Lcp2	-0.99
H2-Aa	-0.88
Pik3cg	-0.84
H2-Eb1	-0.83
Nfatc2	-0.79
Lyn	-0.77
H2-Ab1	-0.76

Antigen Presentation	
Gene	LogFC
Gm11127	-2.02
H2-Aa	-0.88
H2-Eb1	-0.83
Cd74	-0.83
H2-Ab1	-0.76

Leukocyte Extravasation	
Gene	LogFC
Nox1	-5.76
Itgal	-1.98
Prkcb	-1.93
Rasgrp1	-1.83
Arhgap4	-1.50
Rac2	-1.30
Spn	-1.24
Pik3cg	-0.84
Ncf1	-0.77
Itgam	-0.72

FCɣ Recep-Med Phag	
Gene	LogFC
Prkcb	-1.93
Rac2	-1.30
Fyb	-1.28
Pld4	-1.14
Lcp2	-0.99
Hck	-0.97
Pik3cg	-0.84
Lyn	-0.77
Ncf1	-0.77

Neuro-Inflammation Sig	
Gene	LogFC
Nox1	-5.76
Cxcl10	-4.52
Il10	-3.52
Gm11127	-2.02
Grin1	-1.91
Cd86	-1.68
Tlr1	-1.36
Irak3	-1.18
Tlr9	-1.04
H2-Aa	-0.88
Pik3cg	-0.84
H2-Eb1	-0.83
Nfatc2	-0.79
Ncf1	-0.77
H2-Ab1	-0.76
Birc3	-0.62

Comm Btw Immune cells	
Gene	LogFC
Cxcl10	-4.52
Il10	-3.52
Cd79b	-3.26
Gm11127	-2.02
Ighm	-2.00
Cd86	-1.68
Tlr1	-1.36
Ccl3	-1.18
Ccl9	-1.11
Tlr9	-1.04
Cd83	-1.00
H2-Eb1	-0.83

Supplementary Table 2: Genes within IPA pathways in LXRα S196A versus WT from plaque T cells

Cholesterol Bios. P.	
Gene	LogFC
Acat3	4.10
Idi2	3.07
Sqle	1.73
Tm7sf2	1.51
Msmo1	1.19
Ggps1	1.14
Acat1	1.03

Apelin Liver S.	
Gene	LogFC
Col18a1	4.39
Pdgfrb	4.29
Col1a2	3.61
Aplnr	2.55
Col3a1	2.13
Col1a1	1.72

GP6 Signaling	
Gene	LogFC
Col5a2	5.76
Lama2	4.87
Col18a1	4.39
Col6a1	3.94
Col1a2	3.61
Col4a1	3.60
Lama4	2.85
Col4a6	2.27
Col15a1	2.16
Col3a1	2.13
Col1a1	1.72
Lamb3	1.23
Rasgrp2	1.11

Wnt/β-catenin Sign.	
Gene	LogFC
Wnt10b	6.45
Sox6	5.43
Wnt3	5.23
Hnf1a	5.04
Wnt16	4.86
Sox17	3.74
Sox9	3.46
Tgfbr3	2.71
Tgfb2	2.54
Sox1	2.30
Cdh5	2.29
Rarb	2.03
Fzd1	1.87
Ppp2r3a	1.83
Lef1	1.80
Ppp2r5b	1.68
Gja1	1.21

ILK Signaling	
Gene	LogFC
Myh11	4.67
Myh14	3.82
Vegfc	3.41
Lims2	3.33
Myl9	2.96
Fblim1	2.63
Rac3	2.40
Fermt2	2.08
Rhoj	2.07
Snai2	1.99
Ppp2r3a	1.83
Lef1	1.80
Ppp2r5b	1.68
Rps6ka5	1.62
Actn2	1.53
Actn1	1.48
Casp3	1.28
Rnd1	1.03

Reg. EMT Transition	
Gene	LogFC
Wnt10b	6.45
Wnt3	5.23
Hnf1a	5.04
Wnt16	4.86
Lox	2.91
Aph1a	2.02
Snai2	1.99
Fzd1	1.87
Lef1	1.80
Brca1	1.67

Gαs Signaling	
Gene	LogFC
Gng11	-6.58
Cngb3	-6.21
Prkar1b	-6.06
Pth1r	-5.49
Rapgef3	-5.37
Adcy5	-5.37
Creb3l4	-5.20
Creb3l3	-5.13
Chrm3	-4.45
Gnb3	-4.00
Adora2a	-2.77
Ptgir	-2.54
Gnb5	-2.05
Adcy9	-2.00
Ryr1	-1.43
Prkaca	-1.29
Creb3	-1.07
Mapk3	-1.04

Axonal Guidance Sign.	
Gene	LogFC
Efna2	-8.24
Pdgfd	-7.79
Adamts13	-7.36
Gng11	-6.58
Adam21	-6.44
Prkar1b	-6.06
Wnt9a	-6.02
Adamdec1	-5.99
Myl2	-5.89
Erbb2	-5.88
Mmp2	-5.82
Plxnb3	-5.70
Rhod	-5.63
Opn1sw	-5.50
Bmp10	-5.44
Myl7	-5.35
Pik3r3	-5.28
Myl6b	-5.11
Adam30	-4.93
Gm4787	-4.72
Mmp27	-4.41
Mmp28	-4.40
Lrrc4c	-4.39
Mmp23	-4.27
Mmp20	-4.23
Wnt8b	-4.07
Gnb3	-4.00
Prkcz	-3.88
Prkcg	-3.79
Adam32	-3.75
Mmp8	-3.68
Ablim2	-3.37
Fzd4	-3.15
Kif7	-2.76
Pik3r2	-2.71
Unc5b	-2.67
Ablim3	-2.51
Sema6b	-2.22
Rgs3	-2.18
Pdgfa	-2.16
Ptch1	-2.15
Glis2	-2.13
Gnb5	-2.05
Gna15	-1.99
Pik3c3	-1.93
Sema6d	-1.61
Chmp1a	-1.61
Ace	-1.50
Rras	-1.40
Crkl	-1.38
Klc1	-1.32
Prkaca	-1.29
Limk2	-1.28
Sufu	-1.09
Itsn1	-1.08
Mapk3	-1.04
Mmp14	-1.01

Glutathione-Med. Detox	
Gene	LogFC
Gstm5	-7.40
Gstm4	-6.59
Gstp2	-5.43
Mgst1	-3.05
Lancl1	-1.82
Ggh	-1.18
Gsto1	-1.08
Gstz1	-1.03

Xenobiotic Met. Sign.	
Gene	LogFC
Gal3st2	-8.74
Gstm5	-7.40
Hs3st3a1	-7.23
Nos2	-6.93
Gstm4	-6.59
Gstp2	-5.43
Pik3r3	-5.28
Aldh5a1	-5.19
Hs6st2	-4.79
Prkcz	-3.88
Prkcg	-3.79
Cyp1b1	-3.05
Mgst1	-3.05
Map3k13	-3.02
Pik3r2	-2.71
Chst2	-2.69
Map3k10	-2.69
Aldh3b1	-2.58
Ugt1a7c	-2.38
Mgmt	-2.23
Camk2a	-1.97
Map2k6	-1.95
Pik3c3	-1.93
Chst1	-1.91
Aldh6a1	-1.68
Mapk7	-1.61
Hs3st4	-1.57
Abcc3	-1.54
Aip	-1.44
Ndst2	-1.43
Rras	-1.40
Fmo5	-1.37
Aldh1a2	-1.25
Camk1	-1.20
Map3k11	-1.14
Gsto1	-1.08
Mapk3	-1.04
Gstz1	-1.03
Map2k4	-1.02
Aldh9a1	-1.01

Oleate Biosynthesis	
Gene	LogFC
Ufsp1	-5.44
Fads2	-4.18
Cyb5a	-2.28
Fads1	-1.86
Aldh6a1	-1.68
Ptprt	-1.65

Colorectal Cancer Met.	
Gene	LogFC
Nos2	-6.93
Gng11	-6.58
Prkar1b	-6.06
Wnt9a	-6.02
Mmp2	-5.82
Ptger3	-5.74
Rhod	-5.63
Adcy5	-5.37
Pik3r3	-5.28
Rhov	-5.28
Mmp27	-4.41
Mmp28	-4.40
Mmp23	-4.27
Mmp20	-4.23
Wnt8b	-4.07
Gnb3	-4.00
Mapk10	-3.82
Mmp8	-3.68
Tlr12	-3.52
Fzd4	-3.15
Tlr6	-2.93
Pik3r2	-2.71
Gnb5	-2.05
Adcy9	-2.00
Tlr5	-1.95
Pik3c3	-1.93
Mmp19	-1.59
Rras	-1.40
Tcf7l2	-1.29
Prkaca	-1.29
Casp9	-1.15
Rhot2	-1.11
Tlr11	-1.10
Egfr	-1.08
Smad2	-1.08
Trp53	-1.05
Mapk3	-1.04
Map2k4	-1.02
Mmp14	-1.01

Supplementary Table 3: Genes within IPA pathways in LXRα S196A versus WT from pWAT FBC

Glutamate Degradation	
Gene	LogFC
Abat	2.21
Suclg2	1.61

Glutathione Redox R.	
Gene	LogFC
Gpx5	7.24
Gstp-ps	5.88
Gpx2	4.06

Apelin Adipocyte Sign.	
Gene	LogFC
Gpx5	7.24
Gstp-ps	5.88
Gpx2	4.06
Mapk4	2.76
Adcy6	2.14

Matrix Metallop. Inh.	
Gene	LogFC
Mmp1a	5.96
Mmp28	3.39
Mmp15	1.34

GABA Recept. Sign.	
Gene	LogFC
Abat	2.21
Adcy6	2.14
Cacna1c	1.78
Kcnq3	1.16

Lip./Lip. Rafts in Influ.	
Gene	LogFC
Ifng	4.38
Ifnk	3.90

Endothelin-1 Sign.	
Gene	LogFC
Pla2g2e	-5.97
Gucy2c	-3.90
Gnai1	-2.94
Abhd3	-2.75
Casq1	-2.67
Pla2r1	-1.01

Myc-Med. Apoptosis	
Gene	LogFC
Fasl	-4.96
Cdkn2a	-4.58
Prkag1	-1.07

Wnt/β-catenin Sign.	
Gene	LogFC
Hnf1a	-5.86
Cdkn2a	-4.58
Wnt5a	-2.28
Ppm1l	-1.67
Lrp5	-1.06
Ppard	-1.02

Fatty Acid Oxidation	
Gene	LogFC
Bco2	-1.85
Aldh3b3	-1.74

Atherosclerosis Sign.	
Gene	LogFC
Pla2g2e	-5.97
Clu	-4.18
Alox15	-3.86
Abhd3	-2.75
Apom	-2.40
Pla2r1	-1.01

Eicosanoid Signaling	
Gene	LogFC
Pla2g2e	-5.97
Ptgis	-5.69
Alox15	-3.86
Abhd3	-2.75
Pla2r1	-1.01

Supplementary Table 4: Genes within IPA pathways in LXRα S196A versus WT from pWAT FB

Hematopoiesis fr. PSC	
Gene	LogFC
Il5	5.80
Csf2	3.66
Il12b	3.15
Cd3g	1.77

Hematopoiesis fr. MSC	
Gene	LogFC
Il5	5.80
Csf2	3.66

Cytokine Prod. in Mac.	
Gene	LogFC
Csf2	3.66
Il12b	3.15

Glycolysis	
Gene	LogFC
Aldob	4.43
Eno4	1.18

Gluconeogenesis	
Gene	LogFC
Aldob	4.43
Eno4	1.18

Pyruvate Fermentation	
Gene	LogFC
Ldhc	4.53

PI3K/AKT	
Gene	LogFC
Itga3	-2.94
Il21r	-1.41
Il2rg	-1.11
Ikbke	-1.07

IL7 Signaling	
Gene	LogFC
Dntt	-5.87
Ighg2c	-5.59
Il2rg	-1.11

Mol. Mech. of Cancer	
Gene	LogFC
Wnt8b	-3.65
Prkar1b	-3.03
Itga3	-2.94
Smad9	-2.35
Wnt5a	-2.02
Bmp8b	-1.90
E2f8	-1.22

Hepatic Fibrosis Sign.	
Gene	LogFC
Wnt8b	-3.65
Prkar1b	-3.03
Itga3	-2.94
Ednra	-2.52
Spp1	-2.39
Wnt5a	-2.02
Ikbke	-1.07

NANOG in ESC Pluripot.	
Gene	LogFC
Wnt8b	-3.65
Smad9	-2.35
Wnt5a	-2.02
Bmp8b	-1.90

Primary Immunodef. S.	
Gene	LogFC
Cd19	-6.10
Ada	-2.18
Il2rg	-1.11

Supplementary Table 5: Genes within IPA pathways in LXRα S196A versus WT from pWAT T cells

Th1 and Th2 Activation	
Gene	LogFC
Il4	3.54
Ccr3	3.08
Acvr1c	2.98
Klrc1	2.16
Cd40lg	2.07
Icos	1.58
Il2rb	1.40
Cd3g	1.40
Ifng	1.38
Cd28	1.30
Cd3d	1.28
Il18r1	1.25

Th1	
Gene	LogFC
Il4	3.54
Klrc1	2.16
Cd40lg	2.07
Icos	1.58
Cd3g	1.40
Ifng	1.38
Cd28	1.30
Cd3d	1.28
Il18r1	1.25

Th2	
Gene	LogFC
Il4	3.54
Ccr3	3.08
Acvr1c	2.98
Icos	1.58
Il2rb	1.40
Cd3g	1.40
Ifng	1.38
Cd28	1.30
Cd3d	1.28

Crosstalk btw DC & NKC	
Gene	LogFC
Il4	3.54
Csf2	2.79
Cd40lg	2.07
Camk2b	2.03
Il2rb	1.40
Ifng	1.38
Cd28	1.30

T Helper Cell Different.	
Gene	LogFC
Il4	3.54
Cd40lg	2.07
Icos	1.58
Ifng	1.38
Cd28	1.30
Il18r1	1.25

iCos iCosL Sign. In T H.	
Gene	LogFC
Cd40lg	2.07
Camk2b	2.03
Icos	1.58
Il2rb	1.40
Cd3g	1.40
Cd28	1.30
Cd3d	1.28

Antigen Presentation	
Gene	LogFC
Psmb5	-1.67
Ciita	-1.26
H2-DMb1	-1.08
H2-Eb1	-1.06

Atherosclerosis Sign.	
Gene	LogFC
Rbp4	-6.40
Mmp13	-2.59
Ccl12	-2.28
Lyz1	-2.20
Pdgfa	-2.18
Lpl	-1.56
Apoe	-1.15
Pcyox1	-1.13

VDR/RXR Activation	
Gene	LogFC
Igfbp6	-3.17
Pdgfa	-2.18
Hr	-2.06
Vdr	-1.51
Cebpa	-1.36
Ppard	-1.27
Cd14	-1.01

FCγ Rec-Med. Phagocyt.	
Gene	LogFC
Src	-2.78
Mapk3	-1.63
Fcgr3	-1.22
Ncf1	-1.21
Fgr	-1.20
Dock1	-1.17
Syk	-1.09
Hck	-1.07

LXR/RXR Activation	
Gene	LogFC
Rbp4	-6.40
Ccl12	-2.28
Lyz1	-2.20
Lpl	-1.56
Il1rl2	-1.55
Apoe	-1.15
Pcyox1	-1.13
Pltp	-1.08
Scd1	-1.04
Cd14	-1.01

Complement System	
Gene	LogFC
C6	-3.01
C1qc	-2.07
C5ar1	-1.93
Cr2	-1.75
C1qb	-1.64
C1qa	-1.49
C3ar1	-1.15

Supplementary Table 6: Cytokines differentially expressed in LXRα WT versus S196A macrophages and T cells from plaque and pWAT

Cyrokine gene symbols	
**Human**	**Mouse**
IL1B	Il1b
IL27	Il27
IFNG	Ifng
IL1A	Il1a
TNF	Tnf
ICAM1	Icam1
IL6	Il6
CXCL1	
CCL4	Ccl4
CXCL2	Cxcl1
CCL3	Ccl3
CXCL9	Cxcl9
CXCL10	Cxcl10
CCL2	Ccl12
CCL5	Ccl5
TIMP1	Timp1
IL4	Il4
IL13	Il13
IL10	Il10
CCL1	Ccl1
IL1RN	Il1rn

Plaque CD68+ cells S196A vs WT

Gene	logFC	logCPM	PValue	FDR	IN CYTOKINE LIST
**Cxcl10**	-4.5181803	6.81757319	2.87E-06	0.00145392	TRUE
**Il10**	-3.5216461	5.94293414	0.00727541	0.58578367	TRUE
**Ccl3**	-1.1810085	6.74318444	0.03047074	1	TRUE

Plaque T cells S196A vs WT

Gene	logFC	logCPM	PValue	FDR	IN CYTOKINE LIST
**Il27**	-5.0374382	-0.1602636	1.91E-05	0.00034184	TRUE
* **Il4**	5.33269845	0.96143339	2.40E-05	0.00040759	TRUE
**Ccl1**	-6.28282	-1.3368069	0.00026277	0.00261577	TRUE
**Il10**	-1.2065791	5.94293414	0.00149055	0.00985684	TRUE
**Il13**	-3.5241425	-0.8243496	0.03704799	0.10843988	TRUE

pWAT FBC S196A vs WT

Gene	logFC	logCPM	PValue	FDR	IN CYTOKINE LIST
**Ifng**	4.37547134	3.53356936	4.37E-05	0.00406814	TRUE
**Timp1**	2.14935947	3.18141075	0.00517614	0.07674798	TRUE
**Il1a**	1.79310939	4.82842474	0.01678632	0.15108412	TRUE
**Ccl1**	3.445453	-1.3368069	0.02049116	0.16893682	TRUE
**Cxcl9**	0.61220416	6.43347952	0.02863999	0.2031894	TRUE
**Cxcl10**	0.60291901	6.81757319	0.04435914	0.25779763	TRUE

pWAT FB S196A vs WT

Gene	logFC	logCPM	PValue	FDR	IN CYTOKINE LIST
**Il1rn**	-0.6892813	7.64877318	0.01738561	0.18644874	TRUE
**Il13**	2.67056433	-0.8243496	0.04863024	0.30770513	TRUE

pWAT T cells S196A vs WT

Gene	logFC	logCPM	PValue	FDR	IN CYTOKINE LIST
**Ccl12**	-2.2827551	6.25617007	0.00018375	0.00548422	TRUE
**Timp1**	-2.839394	3.18141075	0.00179274	0.02606055	TRUE
**Ccl3**	-0.9298593	6.74318444	0.00206309	0.02866657	TRUE
* **Il4**	3.54020535	0.96143339	0.0029395	0.03689565	TRUE
**Ifng**	1.38282339	3.53356936	0.00354547	0.04161017	TRUE
**Il1rn**	-0.899455	7.64877318	0.00723997	0.06576119	TRUE
**Ccl4**	-0.6207519	6.34774006	0.04986694	0.2099485	TRUE

* Il4 is induced in plaque and pWAT Tcells
