# Supplementary material for: Inhibiting LXRα phosphorylation in hematopoietic cells reduces inflammation and attenuates atherosclerosis and obesity in mice
Source: Commun Biol. 2021 Mar 26;4:420. doi: 10.1038/s42003-021-01925-5 (PMC7997930; doi:10.1038/s42003-021-01925-5)
Supplement: Supplementary file 2 — Description of Additional Supplementary Files [file 42003_2021_1925_MOESM2_ESM.pdf]

Description of Additional Supplementary Files

File name: Supplementary Data 1
Description: Source data for Figures 1-8, and Supplementary Figures 1 and 2.

File name: Supplementary Data 2
Description: Genes differentially expressed in LXRα S196A versus WT from plaque CD68+ cells.

File name: Supplementary Data 3
Description: Genes differentially expressed in LXRα S196A versus WT from plaque T cells.

File name: Supplementary Data 4
Description: Genes differentially expressed in pWAT ATMs (FBC and FB) from LXRα WT and S196A.

File name: Supplementary Data 5
Description: Genes differentially expressed in pWAT T cells from LXRα WT and S196A.
