# Supplementary material for: Inhibiting LXRα phosphorylation in hematopoietic cells reduces inflammation and attenuates atherosclerosis and obesity in mice
Source: Commun Biol. 2021 Mar 26;4:420. doi: 10.1038/s42003-021-01925-5 (PMC7997930; doi:10.1038/s42003-021-01925-5)
Supplement: Supplementary file 8 — Reporting Summary [file 42003_2021_1925_MOESM8_ESM.pdf]

nature research

Corresponding author(s): Edward A. Fisher & Michael Garabedian

Last updated by author(s): 02/09/2021

Reporting Summary

Nature Research wishes to improve the reproducibility of the work that we publish. This form provides structure for consistency and transparency in reporting. For further information on Nature Research policies, seeAuthors & Referees and theEditorial Policy Checklist .

Statistics

For all statistical analyses, confirm that the following items are present in the figure legend, table legend, main text, or Methods section.

n/a	Confirmed	
☐	☒	The exact sample size (*n*) for each experimental group/condition, given as a discrete number and unit of measurement
☐	☒	A statement on whether measurements were taken from distinct samples or whether the same sample was measured repeatedly
☐	☒	The statistical test(s) used AND whether they are one- or two-sided<br>*Only common tests should be described solely by name; describe more complex techniques in the Methods section.*
☒	☐	A description of all covariates tested
☐	☒	A description of any assumptions or corrections, such as tests of normality and adjustment for multiple comparisons
☐	☒	A full description of the statistical parameters including central tendency (e.g. means) or other basic estimates (e.g. regression coefficient) AND variation (e.g. standard deviation) or associated estimates of uncertainty (e.g. confidence intervals)
☐	☒	For null hypothesis testing, the test statistic (e.g. *F*, *t*, *r*) with confidence intervals, effect sizes, degrees of freedom and *P* value noted<br>*Give P values as exact values whenever suitable.*
☒	☐	For Bayesian analysis, information on the choice of priors and Markov chain Monte Carlo settings
☒	☐	For hierarchical and complex designs, identification of the appropriate level for tests and full reporting of outcomes
☒	☐	Estimates of effect sizes (e.g. Cohen's *d*, Pearson's *r*), indicating how they were calculated

Our web collection on statistics for biologists contains articles on many of the points above.

Software and code

Policy information about availability of computer code

Data collection	N/AWe
Data analysis	Statistical testing was completed using Prism 8.0.2 for PC.

For manuscripts utilizing custom algorithms or software that are central to the research but not yet described in published literature, software must be made available to editors/reviewers. We strongly encourage code deposition in a community repository (e.g. GitHub). See the Nature Research guidelines for submitting code & software for further information.

Data

Policy information about availability of data

All manuscripts must include a data availability statement. This statement should provide the following information, where applicable:

- Accession codes, unique identifiers, or web links for publicly available datasets
- A list of figures that have associated raw data
- A description of any restrictions on data availability

We are providing with this manuscript a document with raw data associated to any graph bars, and the full western blots as the last supplementary figure.

Field-specific reporting

Please select the one below that is the best fit for your research. If you are not sure, read the appropriate sections before making your selection.

☒ Life sciences ☐ Behavioural & social sciences ☐ Ecological, evolutionary & environmental sciences

For a reference copy of the document with all sections, see nature.com/documents/nr-reporting-summary-flat.pdf

Life sciences study design

All studies must disclose on these points even when the disclosure is negative.

Sample size	A power analysis showed that for a 2-fold change in a parameter, with a standard deviation up to 60% of the mean, there is a 94% probability that 10 mice/group will be enough to detect a difference at a 5% level of significance.
Data exclusions	No data were excluded from the analyses.
Replication	All experiments were reproduced 2-3 times and had high reproducibility.
Randomization	Samples were randomly allocated into experimental groups.
Blinding	Blinding was not possible due to labeling of experimental materials, but is not necessary since the methods used are objective quantifications such as ELISA, flow cytometry and staining quantification.

Reporting for specific materials, systems and methods

We require information from authors about some types of materials, experimental systems and methods used in many studies. Here, indicate whether each material, system or method listed is relevant to your study. If you are not sure if a list item applies to your research, read the appropriate section before selecting a response.

Materials & experimental systems

n/a	Involved in the study	
☐	☒	Antibodies
☐	☒	Eukaryotic cell lines
☒	☐	Palaeontology
☐	☒	Animals and other organisms
☒	☐	Human research participants
☒	☐	Clinical data

Methods

n/a	Involved in the study	
☒	☐	ChIP-seq
☐	☒	Flow cytometry
☒	☐	MRI-based neuroimaging

Antibodies

Antibodies used	The antibodies used for immunochemistry and immunofluorescence were as follows: CD68 (1:250, MCA1957, Biorad), cleaved caspase 3 (1:100, 9664, Cell signaling), Ki67 (1:100, ab1667, abcam), SMaA-AF488 (1:100, 53-9760-82, Invitrogen), UCP1 (1:300, ab23841, abcam), F4/80 (1:200, MCA497RT, Biorad), Clec4f (1:50, MAB2784-SP, R&D Systems).<br>The antibodies used for flow cytometry were as follows: Percp/Cy5.5 anti-mouse CD45 (103132, Biolegend), PE anti-mouse CD115 (135505, Biolegend), APC anti-mouse Ly6C/6G (108412, Biolegend), PE/Cy7 anti-mouse CD45 (102114, Biolegend), APC anti-mouse F4/80 (MCA497APC, Biorad), PE anti-mouse CD11b (RM2804, Invitrogen), PE-Texas Red anti-mouse CD11c (MCD11C17, Invitrogen), BV421 anti-mouse IAIE (107631, Biolegend), BUV395 anti-mouse B220 (56793, BD Biosciences), BV786 anti-mouse CD3 (564379, BD Biosciences), PerCP/Cy5.5 anti-mouse CD4 (100540, Biolegend), APC-Cy7 anti-mouse CD8 (557654, BD Biosciences), and BV510 anti-mouse CD25 (740106, BD Biosciences). All have been used at a 1:200 dilution.<br>The antibodies used for western blot were as follows: Adipoq (1:1000, 2789T, Cell Signaling), Fabp4 (1:1000, 2120S, Cell Signaling), Atgl (1:1000, 2439S, Cell Signaling), P-Hsl (Ser563) (1:1000, 4139T, Cell Signaling), Hsl (1:1000,18381T, Cell Signaling), Hsp90 (1:5000, 610419, BD Biosciences), and UCP1 (1:20000, 14670S, Cell Signaling)
Validation	All antibodies were used for applications which are validated on the manufacturer's website. Additionally, antibodies have been tested on negative and positive control.

Eukaryotic cell lines

Policy information about cell lines

Cell line source(s)	The 3T3-L1 (ATCC CL-173) and HEK293 (ATCC CRL-1573) have been purchase from the ATCC.
Authentication	N/A
Mycoplasma contamination	Cell lines tested negative for mycoplasma testing.
Commonly misidentified lines (See ICLAC register)	N/A

Animals and other organisms

Policy information about studies involving animals; ARRIVE guidelines recommended for reporting animal research

Laboratory animals	Male LDLR–/– mice on the C57BL/6 background have been purchased at 8 weeks old from Jackson Laboratory. We have developed a LXRa S196A mouse on C57BL/6 background in collaboration with Dr. Ines Pindea-Torra (University College London). We used wild type (WT) and LXRa S196A C57BL/6 male (6 weeks old) as bone marrow transplant donors. Only males will be used for the experimental subjects, the excess females have been used to maintain and expand colonies more easily.
Wild animals	N/A
Field-collected samples	N/A
Ethics oversight	The NYU School of Medicine has fully accredited animal facilities (AAALAC accreditation since March 2014) under the supervision of full time veterinarians and veterinary technicians who are accessible for consultation at all times. All animal work is approved by the Institutional Animal Care and Use Committee.

Note that full information on the approval of the study protocol must also be provided in the manuscript.

Flow Cytometry

Plots

Confirm that:

- [x] The axis labels state the marker and fluorochrome used (e.g. CD4-FITC).
- [x] The axis scales are clearly visible. Include numbers along axes only for bottom left plot of group (a 'group' is an analysis of identical markers).
- [x] All plots are contour plots with outliers or pseudocolor plots.
- [x] A numerical value for number of cells or percentage (with statistics) is provided.

Methodology

Sample preparation

Flow cytometry on blood: Blood was collected before sacrificing the mice by tail bleeding. Red blood cells were lysed with red blood cells lysis buffer (Sigma-Aldrich). Then cells were resuspended in 2% Fc Block (553142, BD Pharmingen) and blocked for 30 minutes. Cells were stained with Percp/Cy5.5 anti-mouse CD45 (103132, Biolegend), PE anti-mouse CD115 (135505, Biolegend), and APC anti-mouse Ly6C/6G (108412, Biolegend).

Flow cytometry on perigonadal exudates after zymosan injection: C57BL/6 WT or S196A mice were administered zymosan A (Sigma-Aldrich). After 4, 24 or 48 hours mice were sacrificed and peritoneal exudates collected. Red blood cells were lysed with red blood cells lysis buffer (Sigma-Aldrich). Then cells were resuspended in 2% Fc Block (553142, BD Pharmingen) and blocked for 30 minutes. Cells were stained with Percp/Cy5.5 anti-mouse CD45 (103132, Biolegend), PE anti-mouse CD115 (135505, Biolegend), and APC anti-mouse Ly6C/6G (108412, Biolegend).

Immune cells profiling from adipose tissue: Perigonadal fat pads were minced, placed in DMEM supplemented with 10 mg/ml fatty acid-poor BSA, and centrifuged at 1000 × g for 10 minutes. A LPS-depleted collagenase mixture (LiberaseTM, Roche Applied Science) at a concentration of 0.03 mg/ml and 50 units/ml DNase I (Sigma) was added to the tissue, and the samples were incubated at 37 °C in a rotating shaker for 45 minutes. Then, the samples were passed through a 70-μm nylon cell strainer (Corning). The suspension was centrifuged at 1000 × g for 10 minutes, and the pelleted cells were collected as the SVC. The floating cells were collected as the adipocytes. The SVC were resuspended in red blood cells lysis buffer (Sigma-Aldrich) and incubated at room temperature for 5 minutes and washed in PBS. SVC were resuspended in 2% Fc Block (553142, BD Pharmingen) and blocked for 30 minutes. Then fluorophore-conjugated primary antibodies were incubated for 30 minutes: PE/Cy7 anti-mouse CD45 (102114, Biolegend), APC anti-mouse F4/80 (MCA497APC, Biorad), PE anti-mouse CD11b (RM2804, Invitrogen), PE-Texas Red anti-mouse CD11c (MCD11C17, Invitrogen), BV421 anti-mouse IAIE (107631, Biolegend), BUV395 anti-mouse B220 (56793, BD Biosciences), BV786 anti-mouse CD3 (564379, BD Biosciences), PerCP/Cy5.5 anti-mouse CD4 (100540, Biolegend), APC-Cy7 anti-mouse CD8 (557654, BD Biosciences), and BV510 anti-mouse CD25 (740106, BD Biosciences) in FACS buffer (1X HBSS (Thermo Fisher Scientific), 2% BSA and 0.5 mM EDTA). After one wash in FACS buffer, cells were resuspended in FACS buffer containing 1 μg/mL DAPI (Invitrogen).

Immune cells profiling from aortas: aortic arches were minced and digested in a LPS-depleted collagenase mixture (LiberaseTM, Roche Applied Science) at a concentration of 0.05 mg/ml, 0.1mg/ml of hyaluronidase (Sigma-Aldrich), 1μM Ca2+ (calcium dichloride, Sigma-Aldrich), and 50 units/ml DNase I (Sigma-Aldrich), and the samples were incubated at 37 °C in a rotating shaker for 15 minutes. The samples were filtered through a 70-μm nylon cell strainer (Corning). The suspension was centrifuged at 1000 × g for 10 minutes, and the pelleted cells were collected, resuspended in red blood cells lysis buffer (Sigma-Aldrich) and incubated at room temperature for 5 minutes and washed in PBS. Aortic suspensions were resuspended in 2% Fc Block (553142, BD Pharmingen) and blocked for 30 minutes. Then fluorophore-conjugated primary antibodies were incubated for 30 minutes: PE/Cy7 anti-mouse CD45 (102114, Biolegend), APC anti-mouse F4/80 (MCA497APC, Biorad), PE anti-mouse CD11b (RM2804, Invitrogen), PE-Texas Red anti-mouse CD11c (MCD11C17, Invitrogen), BV421 anti-mouse IAIE (107631, Biolegend), BUV395 anti-mouse B220 (56793, BD Biosciences), BV786 anti-mouse CD3 (564379, BD Biosciences), PerCP/Cy5.5 anti-mouse CD4 (100540, Biolegend), APC-Cy7 anti-mouse CD8 (557654, BD Biosciences), and BV510 anti-mouse CD25 (740106, BD Biosciences) in FACS buffer (1X HBSS (Thermo Fisher Scientific), 2% BSA and 0.5 mM EDTA). All antibodies have been used at 1:200. After one wash in FACS buffer, cells were resuspended in FACS buffer containing 1 μg/mL DAPI (Invitrogen). Immune cells profiling was performed using a FACSAria II cell sorter (BD Biosciences) and data were analyzed with FlowJo v10. Adipose tissue macrophages (FB and FBC) were sorted and collected.

Instrument	Monocytes and neutrophils were identified using a LSRII analyzer (BD Bioscience) and immune cells profiling/sorting was performed using a FACSAria II cell sorter (BD Biosciences).
Software	Flow cytometry data were analyzed using FlowJo v10.
Cell population abundance	FB and FBC have been sorted: FBC (26.3% for WT and 17.1% for SA) and FB (65.7% for WT and 71.9% for SA). This percentage have been established after gating optimization and analysis.
Gating strategy	Flow cytometry on blood and peritoneal macrophages (Zymosan experiments): SSC-A/FSC-A debris exclusion-> FSC-W/FSC-A single cells-> SSC-A/CD45: CD45+ cells are leukocytes -> CD115/Ly6c/6G: CD115+ are monocytes, Ly6C low & Ly6C high, CD115-/Ly6G+ are neutrophils.<br>Immune cells profiling from adipose tissues and aortas:SSC-A/FSC-A debris exclusion-> FSC-W/FSC-A single cells-> DAPI/FSC-A live cells -> SSC-A/CD45 CD45+cells<br>(A) F4/80/CD45: (1) CD45+F4/80+ are macrophages -> CD11c/CD11b: CD11c+CD11b+ are FBC and CD11c-/CD11b+ are FB, (2) CD45+F4/80- -> CD11b/IAIE are DC, (3) CD45+F4/80- -> CD45/B220 are B cells<br>(B) CD3/CD45: CD3+CD45+ are T cells -> CD4/CD8: CD8+CD4- are killer T cells and CD4+/CD8- cells are CD4+ T cells -> on the CD4 + cells population: CD4/CD25: CD25- are helper T cells and CD25+ are Treg.<br>Positive cells have been identify using single color controls and negative cells have been characterized with samples containing all the antibodies except one (control neg for this color).

☒ Tick this box to confirm that a figure exemplifying the gating strategy is provided in the Supplementary Information.
